# Vertical foraging shifts in Hawaiian forest birds in response to invasive rat removal

**DOI:** 10.1371/journal.pone.0202869

**Published:** 2018-09-24

**Authors:** Erin E. Wilson Rankin, Jessie L. Knowlton, Daniel S. Gruner, David J. Flaspohler, Christian P. Giardina, Devin R. Leopold, Anna Buckardt, William C. Pitt, Tadashi Fukami

**Affiliations:** 1 Department of Entomology, University of Maryland, College Park, Maryland, United States of America; 2 School of Forest Resources and Environmental Science, Michigan Technological University, Houghton, Michigan, United States of America; 3 Institute of Pacific Islands Forestry, United States Department of Agriculture, United States Forest Service, Hilo, Hawai‛i, United States of America; 4 Department of Biology, Stanford University, Stanford, California, United States of America; 5 Smithsonian Conservation Biology Institute, Smithsonian Institution, Front Royal, Virginia, United States of America; Auburn University, UNITED STATES

## Abstract

Worldwide, native species increasingly contend with the interacting stressors of habitat fragmentation and invasive species, yet their combined effects have rarely been examined. Direct negative effects of invasive omnivores are well documented, but the indirect effects of resource competition or those caused by predator avoidance are unknown. Here we isolated and examined the independent and interactive effects of invasive omnivorous Black rats (*Rattus rattus*) and forest fragment size on the interactions between avian predators and their arthropod prey. Our study examines whether invasive omnivores and ecosystem fragment size impact: 1) the vertical distribution of arthropod species composition and abundance, and 2) the vertical profile of foraging behaviors of five native and two non-native bird species found in our study system. We predicted that the reduced edge effects and greater structural complexity and canopy height of larger fragments would limit the total and proportional habitat space frequented by rats and thus limit their impact on both arthropod biomass and birds’ foraging behavior. We experimentally removed invasive omnivorous Black rats across a 100-fold (0.1 to 12 ha) size gradient of forest fragments on Hawai‘i Island, and paired foraging observations of forest passerines with arthropod sampling in the 16 rat-removed and 18 control fragments. Rat removal was associated with shifts in the vertical distribution of arthropod biomass, irrespective of fragment size. Bird foraging behavior mirrored this shift, and the impact of rat removal was greater for birds that primarily eat fruit and insects compared with those that consume nectar. Evidence from this model study system indicates that invasive rats indirectly alter the feeding behavior of native birds, and consequently impact multiple trophic levels. This study suggests that native species can modify their foraging behavior in response to invasive species removal and presumably arrival through behavioral plasticity.

## Introduction

The mechanisms by which invasive species impact native species include direct competition (e.g. [1, 2]), herbivory, predation, and indirect effects such as alteration of ecosystem disturbance regimes or resource availability [3]. While habitat fragmentation and invasive species both have received extensive individual attention (e.g. [4, 5]), their combined effects on trophic interactions have rarely been examined (but see [6]). Forest fragmentation is often associated with an increase in invasive species [7, 8], but predicting resulting impacts on native species, let alone trophic structures of an ecosystem, are hindered by the typically confounded and complicated nature of fragmented study systems. For instance, the greater structural complexity frequently observed in larger forest fragments may extend beyond the habitat volume in which an invasive consumer can forage, thereby allowing native species and their interactions to persist [9–12]. Alternatively, the greater habitat complexity of larger forest fragments may support a higher density of invasive consumer species due to the greater amount of microhabitats and resource abundances, which could amplify their impact on native species [7, 13]. Overall, a generalized understanding of these effects is lacking.

When biological invaders impact species interactions, they alter and can even completely disrupt trophic systems [14, 15]. The most potentially disruptive invasive species are those that impact native species across multiple trophic levels, through direct predation, competition for common resources, or alteration of species’ behavior or environmental conditions [16–20]. For these reasons, an important challenge in ecology is to tease apart the mechanisms responsible for direct and indirect effects of invasive predators on community and ecosystem processes [15, 21–23]. Moreover, while the evolutionary responses of native species to invasive species have been studied (see references in [24]), the plastic, behavioral responses of native species to invasive species are less well understood [25–27].

The impacts of plant and animal invaders on species interactions can be more pronounced on islands, especially isolated oceanic islands that have fewer trophic linkages (e.g., [28]). Many island systems have fewer trophic levels than comparatively sized areas within continents, suggesting that trophic interactions related to invasive species might be more apparent [29]. Isolated island ecosystems also may be vulnerable because of their high levels of endemism [30], lack of evolved defenses common to continental species [29], and low functional redundancy [31]. For example, in Hawai‘i, invasive birds may compete with native honeycreepers [32], but see [33], while invasive wasps compete with native arthropods [34] and birds [35] and disrupt native plant-pollinator mutualisms [36].

Invasive rats (*Rattus* spp.) have been introduced to most of the world’s oceanic islands where they have directly or indirectly contributed to declines in native species populations [37–39]. For example, the omnivorous Black rat (*Rattus rattus*), an adept tree climber, consumes a diversity of foods including seeds, fruits, arthropods, carrion, bird eggs and nestlings [40–43]. As a result, rats can have profound effects on multiple trophic pathways [23], and numerous studies provide examples of their wide-ranging effects on island ecosystems [44–50]. Theory indicates that coexistence of species can be facilitated by niche differentiation [51], which may involve resource partitioning [52], or spatial and or temporal avoidance mechanisms [53, 54]. Direct negative effects of Black rats on native birds via predation in the ground and shrub layers have been documented in the Hawaiian Islands (e.g., [40, 55]), but the indirect effects of resource competition with rats or those caused by predator avoidance are unknown. Fragments larger in area are also larger in habitat volume, and they typically produce more fruit and arthropod resources than smaller forest fragments [56].

The overarching goal of this research was to isolate and examine the independent and interactive effects of invasive omnivorous Black rats and forest fragment size on the interactions between avian predators and their arthropod prey. Our two main study questions were whether invasive omnivores and forest fragment size impact: 1) the vertical distribution of arthropod species composition and abundance, and 2) the vertical profile of foraging behaviors of five native and two non-native bird species found in our study system. Based upon these questions and observations that black rats rarely climbed above 12 m in our forests, we predicted that: 1) the reduced edge effects and greater canopy height of larger fragments (due to lessened edge effects) [57] will limit the proportional habitat space frequented by rats and thus limit their impact on both arthropod biomass and birds’ foraging behavior in the canopy; 2) the greater impact of invasive rats in small fragments (<1 ha) will result in larger foraging niche shifts by birds to avoid rats in the small fragments; and 3) because of their larger overlap in diet with rats [43], birds that consume relatively more fruit and insects will shift foraging locations to a greater extent in response to rat removal, compared to birds that are consume more nectar.

## Materials and methods

### Study site and rat removal

**Fig 1:**
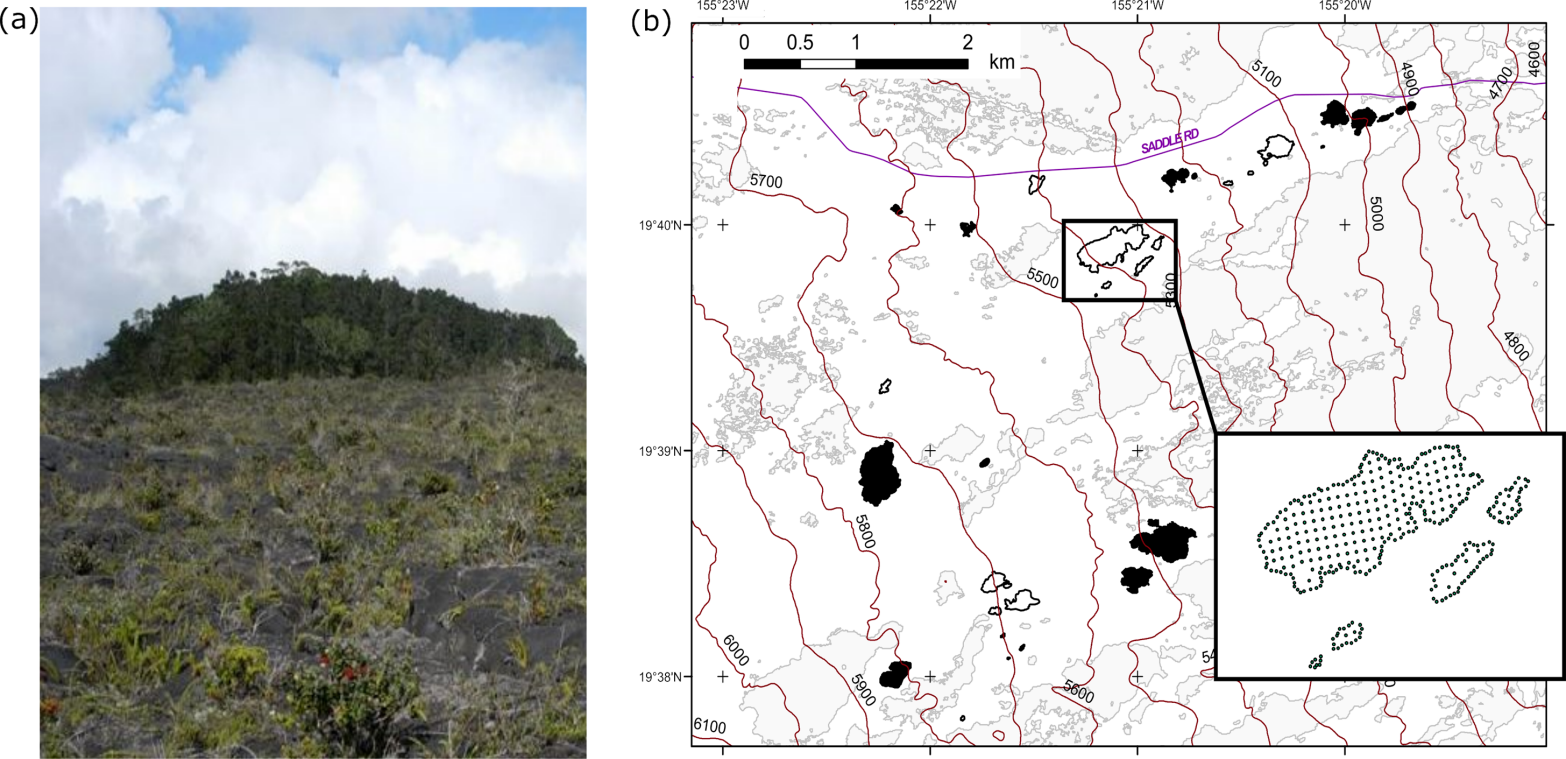
Kīpuka study system on Hawai‘i. (A) A kīpuka (background), which stands out from the small and sparse shrubs growing in the surrounding lava matrix (foreground). (B) The 34 kīpuka (forest fragments) on the NE slope of Mauna Loa, Hawai‘i Island, included in this study (rat-removed = black boundaries, white-filled; untreated (control) = black-filled). Grey shapes in background represent continuous forest and kīpuka not used in this study (LiDAR shape data from Vaughn et al. 2014). Elevation bands are displayed in units of feet above sea level. Inset shows the rat trapping grids installed in five kīpuka, with interior trap stations spaced by 25m and boundary traps 12.5 m apart.

To test our predictions, we carried out our study on Hawai‘i Island on the NE slope of Mauna Loa Volcano (19°40’ N and 155°20’ W, 1470–1790 m elevation), in a ~35 km^2^ landscape consisting of a network of forest fragments (kīpuka) that are remnants of continuous tracts of native forest fragmented by volcanic lava flows in 1855 and 1881. The kīpuka landscape is recognized as a model study system for understanding the effects of long-term fragmentation [57–59]. The system includes kīpuka that occur on approximately 3000- to 5000-years-old lava substrates and consist almost entirely of native plant species, with each kīpuka surrounded by much younger lava substrate of 130–160 years, and variably but lightly colonized by plants. Differences between the kīpuka and lava matrix are visually obvious (Fig 1A). Canopies of the kīpuka forests are dominated by the ʻŌhiʻa (*Metrosideros polymorpha*) tree, with some Koa (*Acacia koa*) trees. The mid-story consists of ‘Ōlapa (*Cheirodendron trigynum*), Pilo (*Coprosma montana*), Kōlea (*Myrsine lessertiana*), Kāwa‘u (*Ilex anomala*) and the tree fern Hapu‘u (*Cibotium glaucum*). The study landscape is managed within the State of Hawai‘i Forest Reserve System, and it has been largely protected from human disturbance since its designation. Several non-native vertebrate species are common in the kīpuka, including Black rats, Polynesian rats (*R*. *exulans*), House mice (*Mus musculus*), Small Asian mongoose (*Herpestes javanicus*), Wild Boar (*Sus scrufa*), Japanese White-eye (*Z*. *japonicus*), Red-billed Leiothrix (*Leiothrix lutea*), and Kalij Pheasant (*Lophura leucomelanos*).

We studied birds and arthropods in 34 of these kīpuka (Fig 1B), ranging in area from 0.07 to 12.37 ha (mean 1.93 ± 3 SD ha). Beginning in June 2011, rats were removed from 16 of the 34 study kīpuka using snap traps baited with peanut butter or coconut as part of a larger study on the interactive effects of predation and ecosystem size on arthropod food webs. Traps were placed in 25-m grids within each kīpuka and every 12.5 m around kīpuka perimeters to deter rat immigration into the rat-removed kīpuka. Traps were checked and re-baited every two weeks for from June 2011-June 2015. To assess the efficacy of our baiting methods, we stationed Black Trakka™ tunnels (10 cm x 10 cm x 50 cm; Gotcha Traps Limited, New Zealand) on 1–2 trees in each kīpuka: one tree at the interior of the kīpuka and one at the edge if kīpuka were > 0.18 ha, and a single tree for the smallest kīpuka. Tracking tunnels were placed on the forest floor, at 6m above the forest floor, and when the tree was sufficiently tall, at 12m. Tracking tunnels were baited and checked prior to rat removal and quarterly thereafter. Although sporadic captures were reported in rat-removed kīpuka (Barney et al. unpublished data), we observed no evidence of rat recolonization during the study period.

### Arthropod biomass: Estimates of prey availability in the canopy

We deployed yellow sticky cards (0.036 m^2^; Seabright Labs, Emeryville, CA) at multiple levels of the canopy to assess the vertical biomass distribution of mobile arthropods. Such methods have commonly been used as proxies for relative arthropod activity and prey availability [60–62]. No single method will produce a perfect measure of actual arthropod prey available to birds, however sticky cards captured many common prey items of passerine birds, excepting lepidopteran larvae, observed at this site and more generally (Table 1). Many of these same taxa also are readily evident from rat diet studies [46, 47]. Although plant-based extraction or enumeration methods may be more appropriate for measuring prey availability for gleaning insectivores (e.g., [63]), sticky cards yield an index of mobile arthropod activity and abundance [64] featuring prey available to both birds and rats, and which provides comparable estimates in any strata in the forest from the ground to upper canopy.

**Table 1:** Breakdown of arthropod dry biomass by taxonomic order averaged over all sticky card traps (N = 290 traps).

Order	Mean biomass (mg)	+/- SE	Proportion of total biomass	References
Acari†	0.308	0.167	0.01%	[79]
Araneae*†	76.593	74.408	1.64%	[79–81]
Coleoptera*†	2.495	0.578	0.05%	[79–82]
Collembola	0.026	0.008	0.00%	
Diplopoda†	10.135	6.471	0.22%	[79]
Diptera*†	3715.444	503.974	79.39%	[79–83]
Hemiptera*†	800.093	150.522	17.10%	[79, 81, 82]
Hymenoptera*†	41.538	24.382	0.89%	[79, 80, 82]
Lepidoptera*†	8.552	1.448	0.18%	[79, 81–83]
Myriapoda	5.333	0.837	0.11%	
Neuroptera*†	15.203	5.423	0.32%	[79, 80]
Psocoptera†	3.749	0.714	0.08%	[79]
Thysanoptera	0.513	0.167	0.01%	

* indicates that this order is commonly detected in avian diets

† indicates that this order has been detected in Hawaiian bird diets [79].

We used these data to test for the effects of rat removal on temporal and spatial variation in arthropod composition and abundance, including pre-removal and post-removal periods, and between untreated and rat-removed kīpuka. In the same trees that hosted the tracking tunnels (see above), we installed a continuous 4mm cord running through the uppermost canopy branches to the tree base. Sticky cards were tied with zip ties to loops in this line and hoisted above the ground to three vertical heights (2m, 6m, and height of highest fork, hereby referred to as maximum height). For trees that were <6m in height, only two cards were used (i.e., 2 m above the ground and maximum height). Mean maximum height of the highest sticky card was 15.7 ± 1.3 m (N = 62 traps per deployment). Sticky card traps were set to track the arthropod community at times that corresponded with foraging observations (see below). After collection, each arthropod was identified to at least order, or to family when feasible, and its body length measured. Arthropod dry biomass was estimated using taxon-specific allometric regression equations developed by Gruner [65]. Biomass data were analyzed on a per sticky card basis.

### Foraging observations

We studied the foraging behavior of seven bird species found in the kīpuka: the Hawai‘i ‘Amakihi (*Chlorodrepanis virens*) and Hawai‘i ‘Elepaio (*Chasiempis sandwichensis*), both of which are native to Hawai‘i and eat a lot of insects; the native ‘Ōma‘o (*Myadestes obscurus*) and non-native Red-billed Leiothrix (*Leiothrix lutea*) and Japanese White-eye (*Zosterops japonicus*), which are known to include lots of fruit in their diets; and the ‘Apapane (*Himatione sanguinea*) and ‘I‘iwi (*Vestiaria coccinea*), which are native and consume a lot of nectar. These diet designations follow Banko & Banko [66], but we acknowledge that all species show flexibility in their diets and that all consume arthropods, especially during the breeding season [66, 67] when need for resources and protein is especially high. To this end, we conducted our arthropod foraging observations during the bird breeding season.

Foraging observations of forest birds were conducted by six observers between dawn and 16:00, one to five days a week, between April 1^st^ and July 20^th^ in both 2012 and 2013 in all 34 study kīpuka. All observers were trained by the same person for one week before data collection, to improve comparability of observations from different observers. Using Google Earth (accessed July 2011), we delineated straight-line transects spaced 40 m apart through each kīpuka that was >1 ha. We then used a compass and GPS to walk these transects at a slow, steady pace (approximately 30 min to traverse 100 m) while looking for foraging birds. For kīpuka <1 ha, we did not use transects but walked systematically through the kīpuka for 30 min to 1 hour depending on kīpuka area. Upon observation of an individual foraging bird, we waited until a second foraging action, and then noted the bird species, the identity of the individual if it had color bands [many birds in the kīpuka were banded in a parallel study, see [68]], a visual estimate of foraging height and canopy height over the bird, and the prey type if it could be identified. Beforehand, all observers practiced height estimations in the kīpuka until they were reliably able to estimate height with 1 m accuracy. To maximize the independence of foraging observations, we attempted to collect only a single observation for each individual bird [69]. However, not all birds were banded, and thus there was a nonzero probability that some individuals were observed more than once. Each kīpuka was sampled twice per year, and kīpuka were surveyed less than once per month to reduce repeated observations of individual birds. To analyze birds’ vertical use of the forest canopy, we calculated the index of canopy utilized for foraging using the formula: max[bird height estimated by observer/tree height]–min[bird height estimated by observer/tree height] for each bird species in each kīpuka in each year, where tree height was the height of the tree in which the bird was observed foraging. This index accounted for relative bird position with variation in canopy height. All animal research was approved by the State of Hawai‘i Department of Land and Natural Resources Division of Forestry and Wildlife (Protected Wildlife permit no. WL13-02), and the Michigan Technological University Institutional Animal Care and Use Committee (IACUC, no. 332879–4).

### Statistical analyses

Full statistical models were designed *a priori* based on the design of the study (i.e., effects of rat removal, kīpuka size, and height in canopy on variables of interest) and our prior research in these systems [57–59, 68, 70]. To evaluate how height where arthropods were trapped, kīpuka size and rat removal influenced arthropod biomass, we used generalized linear mixed models (GLMMs) to model the proportion of arthropod biomass for each tree at each height as the response variable, with natural log(kīpuka area), rat treatment, sticky trap height (m) and sticky trap height by rat treatment interaction as fixed effects, and kīpuka as the random effect. Because the response variable is a proportion, we used a binomial error structure with weights as the sum of all arthropod biomass per tree. Here and in all subsequent GLMM analyses, we used AICc model selection to identify the best model (S1 Table), where models with dAIC<2 are considered equivalent. If there was not one single best model (i.e., Akaike weight > 0.9), then we used a model averaging approach (package MUMIn [71]) to assess the importance of the fixed effects.

We further investigated whether the volume of the canopy forest birds used was affected by bird diet, kīpuka area and rat removal. Our index of canopy utilization by birds for foraging served as the response variable in GLMMs with natural log(kīpuka area), rat treatment, bird diet and bird diet by rat removal interaction as fixed effects, and kīpuka and year as random effects in the full model. We range-standardized the standard deviation for each utilization index estimate (i.e. converted SD to 0 to 1 scale) and used these standardized values as weights. Model averaging was conducted as described above.

To determine how arthropod biomass in the canopy, kīpuka size and rat removal influence bird foraging height, we used GLMMs, where the natural log of foraging height as the response variable, with arthropod biomass per sticky trap, natural log(kīpuka area), rat treatment, bird species, arthropod biomass by rat treatment interaction as fixed effects and kīpuka, year, and observer as random effects in the full model. Because foraging height was the mean of two heights estimated by observers [bird height and the difference in bird distance from the top of the canopy and the maximum tree height], we range-standardized the standard deviation for each canopy estimate (i.e. converted SD to 0 to 1 scale) and used these standardized values as weights. For observations where we had detailed descriptions of the foraging behavior (e.g. glean, sally, etc.), we used a similar approach to foraging height but included either foraging behavior type or horizontal position of foraging as an additional fixed effect.

All statistical analyses were completed in R v. 3.5.0 [72] using GLMMs with the “lmerTest” package [73], which implements “lme4” [74]. The Gaussian error distribution was used unless noted otherwise. In all instances where there were multiple continuous independent variables, we tested for collinearity using functions *kappa*.*mer* and *vif*.*mer* from "mer-utils" and found none (e.g. VIF < 2.5 and kappa < 5) [75]. In no instance was any collinearity detected. For all analyses, the R package “LMERConvenienceFunctions” [76] was used to evaluate and remove outliers (romr.fnc) and for post-hoc testing (mcposthoc.fnc). R package “phia” [77] was used for post-hoc testing of interaction contrasts using t-tests (testInteractions). P-values < 0.05 in post-hoc tests and GLMMs are considered significant and GLMM results of all model averaging are reported in Supplemental Materials (S2–S7 Tables). For each model averaging, we also report the relative variable importance (RVI), which is calculated from model averaged parameter estimate weights. RVI allows us to compare the contribution of each variable to the average model [78].

## Results

### Rat activity

**Fig 2:**
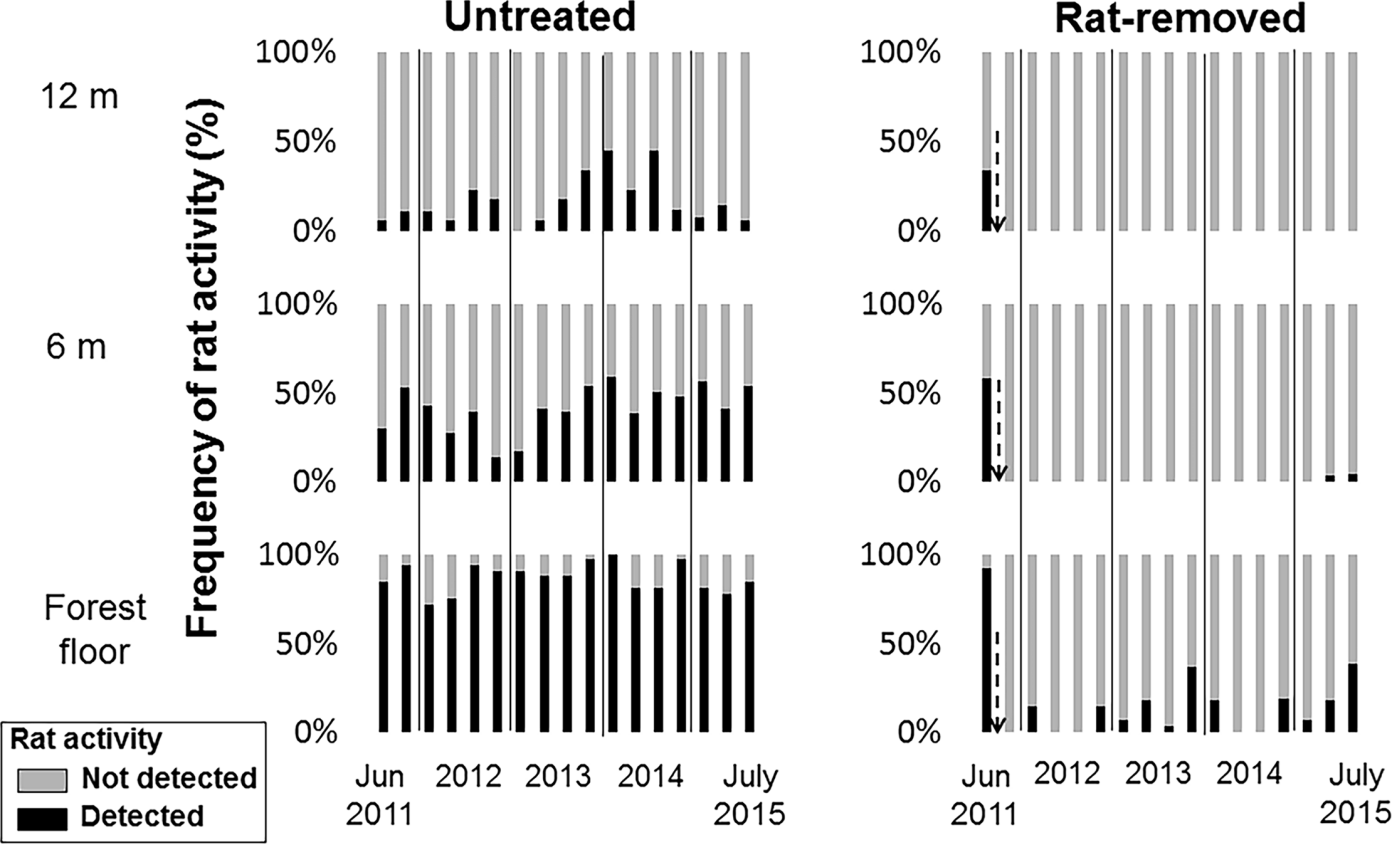
Frequency of rat activity on baited tracking tunnels placed at the forest floor, 6 m and 12 m high in trees in rat removed and control kīpuka.

Before removal efforts began, tracking tunnels placed on trees showed a similar level of rat activity in the future rat-removed and untreated (control) fragments, with more activity on the ground than at 6 or 12 m off the ground (Fig 2). Post-removal tracking tunnel results indicated that our removal efforts were effective, with little sign of rat activity in rat-removed fragments since July 2011 (Fig 2). Tree height was highly correlated with natural log(kīpuka area) (S1 Fig:*Z* = 5.32, *P* < 0.0001) and not with rat treatment (*Z* = 1.29, *P* = 0.20, S2 Table).

### Arthropod biomass

**Fig 3:**
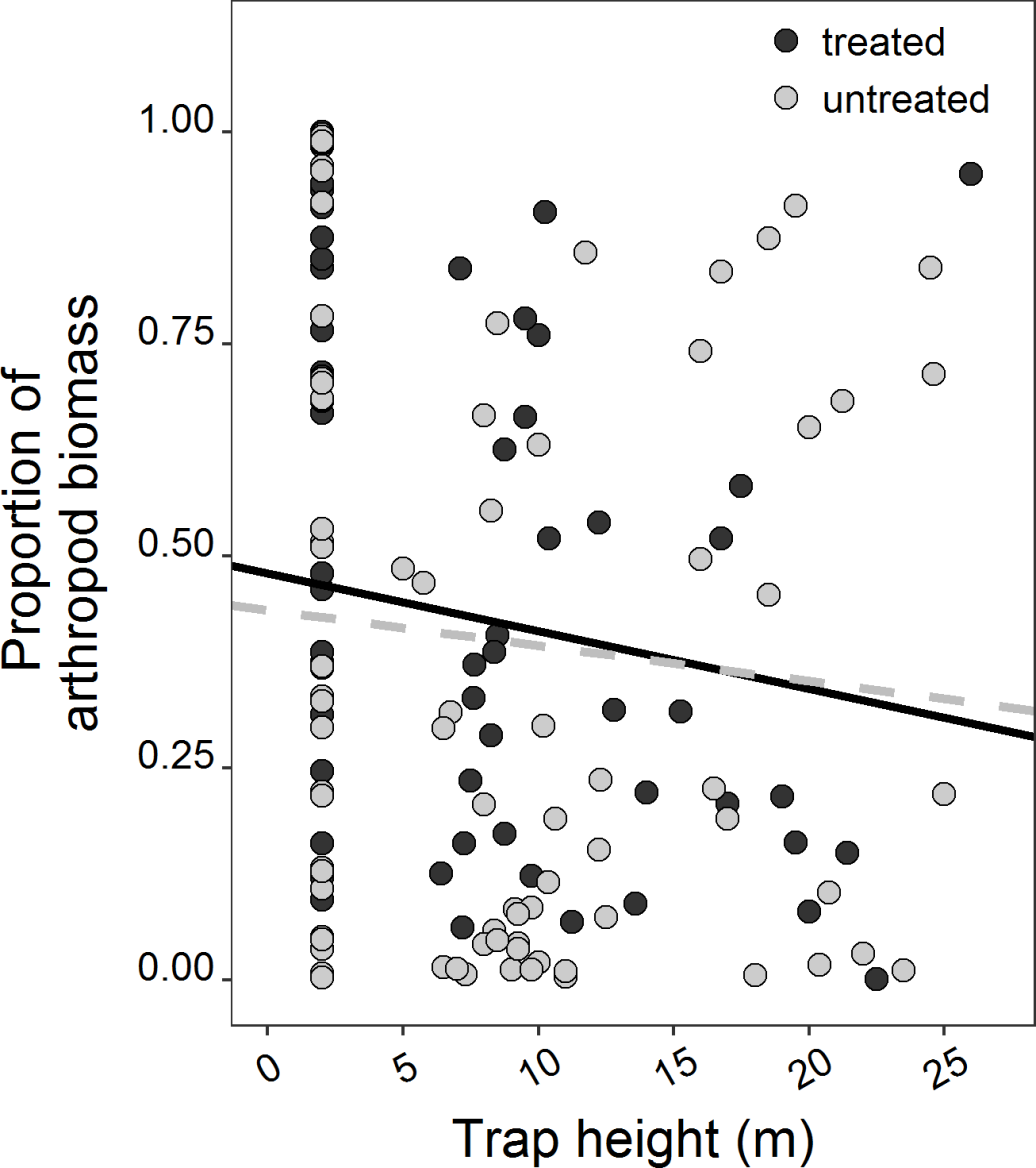
Arthropod biomass distribution across traps by tree.

Arthropod biomass was unevenly distributed in forest canopies (Fig 3), with an average of 65 ± 3% of biomass found at or below a height of 6 m, irrespective of kīpuka size (S3 Table). Vertical distribution patterns interacted significantly with rat treatment (S3 Table: *Z* = 2.67, *P* = 0.0075). This interaction had the highest importance among all variables examined (S3 Table). The traps detected a diverse set of arthropods (Table 1). The orders Diptera and Hemiptera, which are common prey items of Hawaiian birds [66, 79], comprised the bulk of the arthropod biomass (79 and 17%, respectively).

### Bird foraging

**Fig 4:**
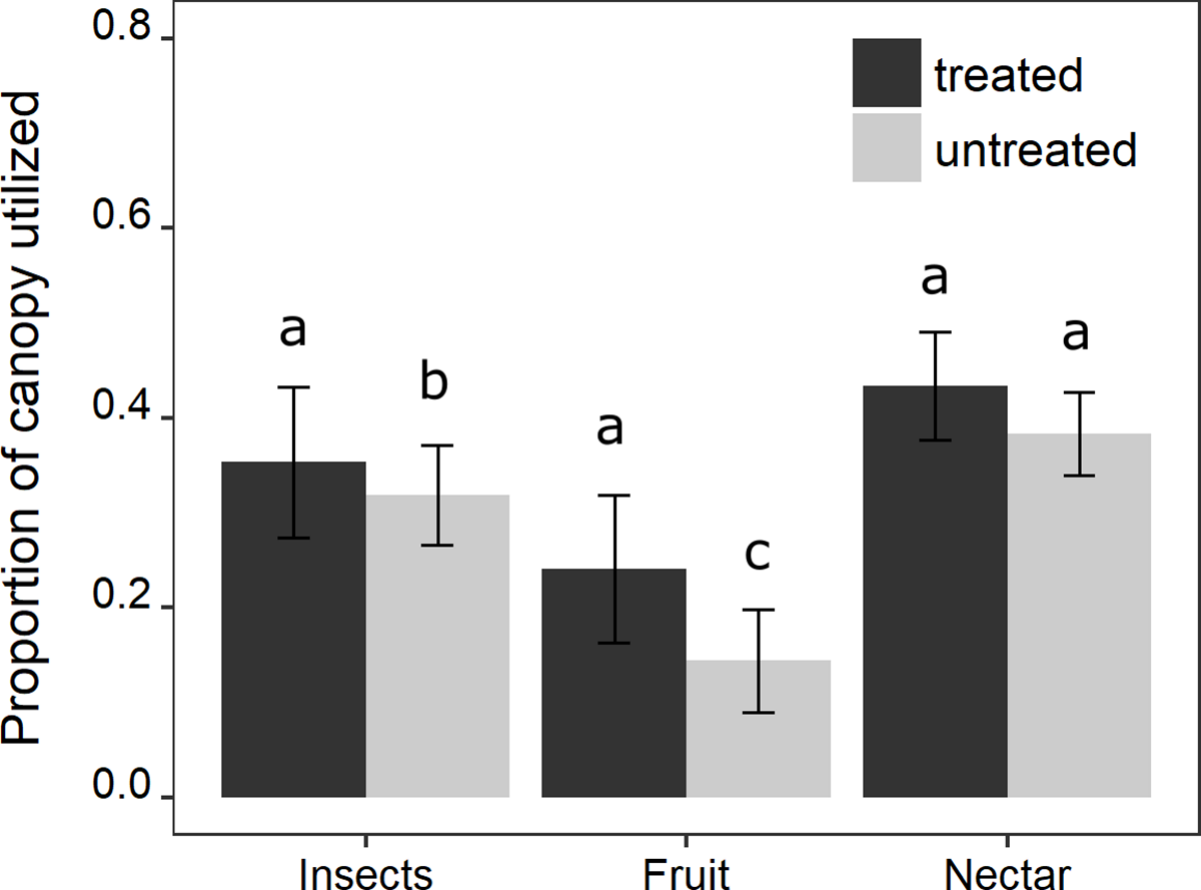
Proportion of canopy utilized by birds by rat treatment and diet components. Birds varied in how much of the available canopy they utilized based on their diet, but all groups utilized more canopy in the absence of rats. Diet categories are relative and not absolute, as all birds consumed arthropods during the breeding season. Different letters indicate significance at p < 0.01.

The proportion of canopy utilized by foraging birds was significantly affected by rat removal; model averaging revealed that diet and rat removal and the rat removal by diet interaction were the most important factors influencing canopy utilization patterns (Fig 4, S4 Table). Across kīpuka, foraging birds utilized the greatest proportion of vertical foraging space (0.45) when rats were removed compared to when rats were present (0.38). While there was an effect of diet (*Z* = 2.481, *P* = 0.0079; *Z* = 5.23, *P* < 0.0001), S4 Table)_,_ all species increased their vertical foraging space with rats removed. Hawai‛i ‘Amakihi and Hawai‘i ‘Elepaio responded similarly to rat removal as did species also known to feed on nectar (‘Apapane and 'I'iwi) (from 0.32 to 0.37 and from 0.40 to 0.45, respectively). ‘Ōma‘o and Japanese white eye, both of which are known to feed on fruit, showed a larger relative increase with rat removal (from 0.14 to 0.37, Fig 4).

**Fig 5:**
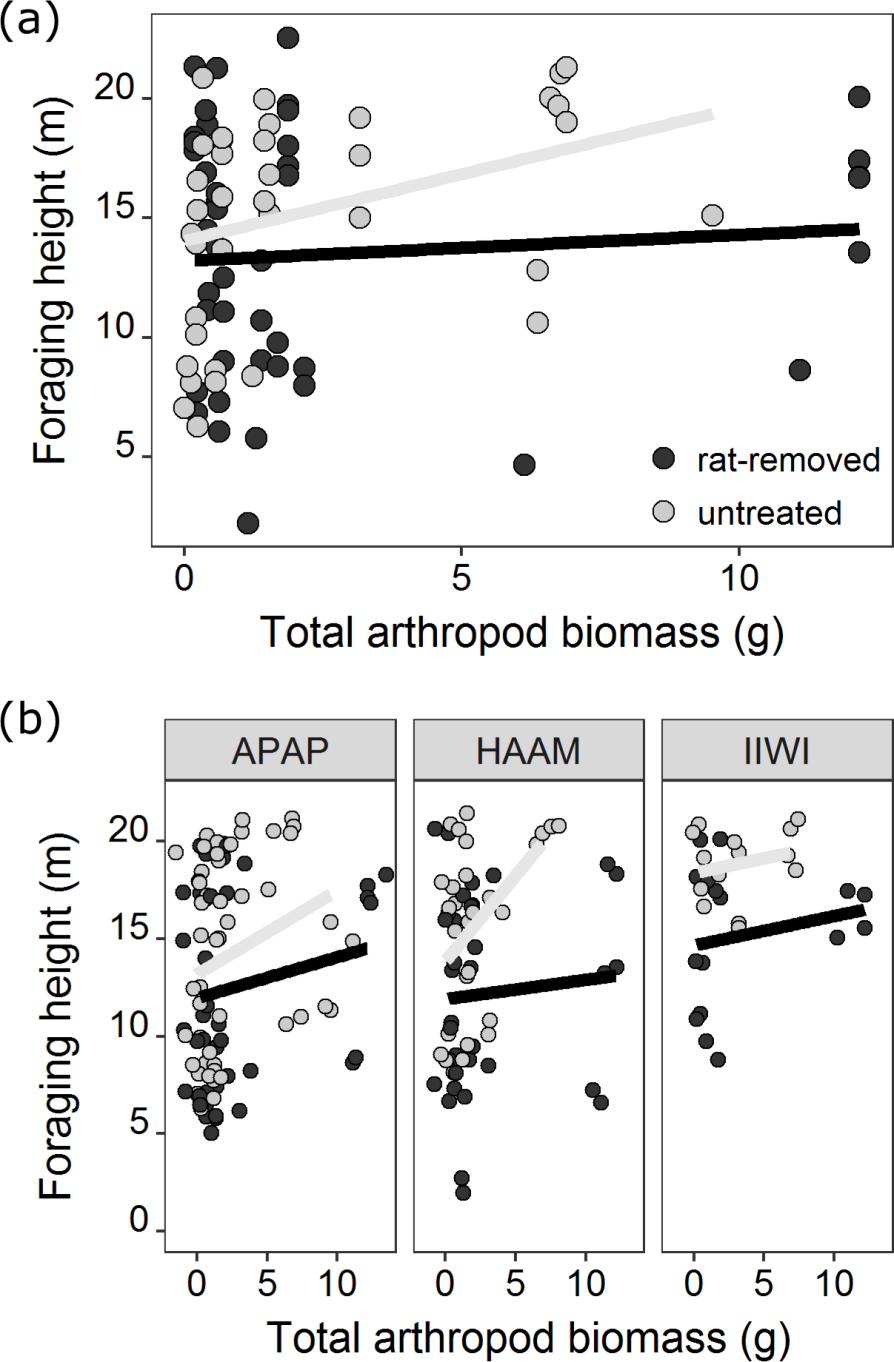
**Mean bird foraging height summarized as a function of arthropod biomass and rat removal treatment for all birds (A), and the three most common species (B).** The open circles represent observations in rat-removed kīpuka and the closed circles represent observations in untreated control kīpuka. Lines represent lines of best model fit for each treatment. APAP: ‘Apapane; HAAM: Hawai‛i ‘Amakihi; IIWI: ‘I‘iwi.

We also assessed how foraging height varied in response to rat removal in this landscape. Foraging heights were correlated with arthropod biomass (*Z* = 12.77, *P* < 0.0001). Birds foraged on average 9.5% lower in rat-removed kīpuka than control kīpuka, but rat removal by itself was relatively less important than other variables in the averaged model (S5 Table). There was, however, a strong interaction effect of rat removal and arthropod biomass: birds foraged at lower mean heights in rat-removed kīpuka (which had more arthropod biomass below 6 m) compared to higher foraging heights in control kīpuka (Fig 5A, S5 Table: arthropod biomass*rat treatment: *P* < 0.0001, RVI = 1). There was also a strong kīpuka area effect (*Z* = 7.72, *P* < 0.0001, RVI = 1), where birds foraged lower in small kīpuka with smaller trees.

Across all kīpuka, birds foraged at different heights depending on species (S5 Table, RVI = 1). Post-hoc tests revealed that Japanese White-eyes foraged higher than Hawai‘i ‘Amakihi, Hawai‘i ‘Elepaio and ‘Ōma‘o, while foraging lower than ‘I‘iwi (S6 Table). ‘Apapane typically foraged higher than the native insectivores and lower than the other native nectarivore, ‘I‘iwi (S6 Table). Focusing just on the most common native species, the strong interaction between rat removal and arthropod biomass is evident, showing that birds followed the arthropod biomass, and tended to forage higher in untreated kīpuka than in treated kīpuka (Fig 5B).

Foraging behavior did not have an effect on the foraging height of Hawaiian forest birds. Across all kīpuka, foraging height was not influenced by specific foraging method employed by the bird (e.g. sally, glean, probe, etc.) (S7 Table: RVI = 0.15) nor was it associated with the horizontal position of foraging (inner, middle or outer part of the branch) (*P* > 0.05, RVI = 0.01) or the density of the foliage (*Z* = 0.60, *P* = 0.56, S6 Table). The type of substrate also had no effect on foraging height (*P* > 0.05, RVI = 0.02).

## Discussion

Our data suggest that invasive rats have indirect effects on how Hawaiian forest birds utilize vertical foraging space. Consistent with our prediction, we linked rat removal to a downward shift in the vertical distribution of arthropod biomass, and bird foraging behavior mirrored this shift, especially in insectivores and frugivores. However, contrary to our prediction, these patterns did not change with kīpuka size. Across the kīpuka size gradient we used in this study, we found that bird foraging height was correlated with arthropod biomass, suggesting that birds track and follow these food sources. Thus, birds appear to be behaviorally plastic in response to the distribution of resources, which in this case are influenced by rats.

### Impacts of invasive rats

Within a tree, we found greater arthropod biomass low in the canopy and a steeper decline in the proportion of arthropod biomass found with increasing trap height when rats were removed from kīpuka, suggesting that rats reduced arthropod biomass in these lower vertical strata. This finding is in accordance with our result that kīpuka rat activity was highest at ground level, was rare to absent above 6 m, and absent above 12 m. Overall, birds foraged higher and used less of the available vertical foraging space in the presence of rats. One potential explanation for this result is that food resources for birds, including arthropods and fruit, were suppressed by rats at lower heights. In addition, the data are consistent with behavioral avoidance of birds to rats as predators. Although all reported depredation of adult Hawaiian forest birds by rats has occurred while the birds slept or sat on nests [42, 84], predator avoidance behavior may occur even in the absence of an immediate threat (e.g., [85]). Recently fledged birds, in contrast, are vulnerable to predation by rats because of their inexperience and less-developed flight capabilities. Our foraging observations were limited to adult foragers. While the direct effects of rats on the reproductive success of Hawaiian birds have been documented [40, 50, 86], our study is the first to indicate indirect impacts of invasive predators on habitat use and foraging of native birds in Hawai‘i.

Past analyses of the diet of Black rats in the Hawaiian Islands show that fruit can make up 50% of rat gut contents, while arthropods make up a smaller fraction of the apparent diet at roughly 15% of gut contents [46, 87]. These analyses would suggest that birds with different relative proportions of nectar, fruit or insects in their diets may respond differently to rat removal. Species known to consume a lot of fruit showed the largest increase in the amount of vertical foraging space used with rat-removal and foraged lower than in control kīpuka, possibly due to the greater diet overlap with rats. These species also commonly include a large proportion of insects in their diet, especially while breeding (e.g., [88]). Rat removal also resulted in lower foraging heights by species known to include a great proportion of insects in their diet, which used more vertical foraging space than in control kīpuka. Species that generally include more nectar in their diets also increased the amount of vertical foraging space used in the absence of rats, but to a lesser degree than the other two primary diet preferences. Rats seem unlikely to deplete nectar resources, however these nectar eating birds supplement their diet with arthropods, especially during the nesting season [66, 67]. If rat removal did boost food resources and/or bird fecundity these kīpuka, some birds may have spilled over into adjacent controls. This spill over could also be an explanation for the different responses of the species with different primary diet preferences, since competitive interactions among them may have driven some birds to forage lower in the trees. A point of caution is that all studies of stomach contents may underestimate the proportion of total diet that are arthropods because many soft-bodied arthropods are difficult to identify after ingestion [89, 90]. Additionally, the actual impact of rats on total abundance of fruits and arthropod resources is not clear from the contents of rat stomachs. For example, while a smaller part of the gut content in terms of percentages, impacts on arthropods may be larger than on fruit if fruit production outweighs insect production per unit of habitat. Finally, our rat trapping caught other non-native species besides rats, albeit less frequently, including mice and mongoose, which may exert some influence on food webs. These mammals also feed on arthropods [87], but effectively do not climb trees [40, 41] and are therefore unlikely to affect arboreal arthropods or bird foraging in the canopy.

Most research on the impacts of invasive species on native island biota has focused on population-level changes or evolutionary adaptation in native species [24]. Several native Hawaiian Hymenoptera declined in numbers apparently due to competition and predation from invasive *Vespula* wasps [34]. Less studied are behavioral changes that some native species may adopt to minimize competition with or predation from invasive species [91]. Here, we present evidence that suggests that invasive rats indirectly alter the foraging behavior, and consequently the realized niche, of native and non-native Hawaiian birds. Few other examples exist of native species displaying behavioral changes in response to the removal of invasive species. On O‘ahu Island, the native O‘ahu ‘Elepaio (*Chasiempis ibidis*) altered its nest-building behavior, favoring higher nesting sites, but this change apparently evolved over time due to predation from invasive rats [92]. In the US Virgin Islands and Puerto Rico, *Anolis* lizards shifted their perching heights and sleeping locations in the presence of invasive rats [27], and in the Caribbean, *Anolis* lizards altered their habitat use in the presence of an invasive predatory *Leiocephalus* lizard [25]. On the Galapagos Islands, *Tropidurus* lizards were less tame and more wary on feral cat-inhabited islands than cat-free islands [26]. Though often overlooked, rapid behavioral response to invasive species’ presence may be important to explain and predict which native species are more vulnerable to biological invasion [but see 54]. The native forest birds that remain on Hawai‘i island may be those that were able to rapidly adapt to the presence of invasive species, whereas many other native birds went extinct soon after human arrival to the islands [44].

### Impacts of forest fragment size

Food resources can play a large role in determining patterns of habitat occupancy for birds in many fragmented systems [93–95], but few studies have demonstrated shifts in foraging behavior based on fragment properties. Surprisingly, only Hawaiian forest bird *foraging height* was positively correlated with kīpuka size, while *canopy utilization* was unaffected by kīpuka size, even though trees are shorter in smaller kīpuka because of greater edge effects [57]. Moreover, birds’ foraging behavior was highly correlated with arthropod biomass. This relationship was strongest for species that eat a lot of insects as well as those that eat a lot of fruit, which are also the two primary diet preferences shown to be most affected by habitat disturbance in tropical regions worldwide [96, 97]. Tropical nectar consumers are usually less sensitive than species with other dietary preferences to fragmentation, possibly because their food resources are more patchily distributed in space and time across the landscape [98]. In Hawai‘i, the nectar consuming ‘I‘iwi and ‘Apapane make wide-ranging movements (average movement distances >1 km over two years) in search of their most common food source, ʻōhiʻa nectar [99], while the relatively more insectivorous Hawai‛i ‘Amakihi and frugivorous ‘Ōma‘o are more sedentary (average movement distances of <1 km over two years) [100]. Larger kīpuka have greater densities of both nectar and fruit resources [56], and likely arthropod densities as well (Gruner et al, unpublished data).

### Interactive impacts of rat removal and forest fragment size

Forest fragment size may modify predator interactions with other species, but these interactions have remained largely understudied [101–103]. As kīpuka area increased, tree height and structural complexity also increased [57]. Because rats forage predominantly below 12 m, we expected that arthropods in the canopy of taller trees, especially in larger kīpuka, would experience reduced predation pressure by rats, with tall canopies functioning as refugia from predators. However, we found that arthropod biomass was affected by rat-removal in all kīpuka, irrespective of size. Our analyses indicated that predators of arthropods are present throughout the canopy in both treatments, although the identity of those predators varied by treatment. This suggests that arthropods were consumed by rats and birds in untreated kīpuka, but mostly experienced predation by birds in treated kīpuka.

Birds appeared to respond to microhabitats where the arthropod biomass was greater rather than to kīpuka area. The interactive effects of Black rats and forest fragment size may have been absent in this landscape because of the scale of rat-removal. None of the kīpuka in this study was more than 500m from another forest patch. While such separation may be sufficient for arthropods to respond to these kīpuka as distinct and independent sites, most bird species likely perceived the kīpuka as part of a landscape mosaic. Supporting this conclusion, we observed a high degree of movement among banded individuals of all bird species in the kīpuka landscape [68].

## Conclusions

Our study explicitly examined interactive effects of habitat fragmentation and invasive species on native fauna. Within the canopy, bird species can adjust behavior in response to the removal of rats, which has implications for understanding and managing for realized niches. Habitat alterations, climate change, novel predators, or competitively dominant invasive or non-native species are among the factors known to cause shifts in species’ realized niches [104]. While many studies have focused on shifts in invasive species’ niches in their new ranges [e.g., 105], here we demonstrated that native species may also modify their realized niche in response to invasive species removals, with potential impacts on multiple trophic levels.

## Supporting information

S1 Fig Height of canopy increases with kīpuka size. Canopy heights were averaged over all foraging observations by kīpuka. Data are shown ± SE. Solid black line indicates line of best fit for treated kīpuka and dotted gray for untreated kīpuka. Canopy height increased with kīpuka size and did not differ between treatments.
(PDF)

S2 Fig Mean foraging height of birds by rat treatment and horizontal foraging position. Foraging height was unaffected by horizontal foraging position of the bird within the canopy. Different letters indicate significance at p < 0.01.
(PDF)

S1 Table GLMM model selection. (PDF)

S2 Table GLMM model averaging results: Kīpuka characteristics and tree height. (PDF)

S3 Table GLMM model averaging results: Proportion of arthropod biomass per trap. (PDF)

S4 Table GLMM model averaging results: Proportion of vertical foraging space occupied (canopy utilization by birds). (PDF)

S5 Table GLMM model averaging results: Foraging heights of Hawaiian forest birds by bird species. (PDF)

S6 Table Post-hoc testing for Species (p-value adjustment method: fdr) of model described in Table D. (PDF)

S7 Table GLMM model averaging results: Foraging behavior effects on foraging height. (PDF)

## References

[1] Preston DL, Henderson JS, Johnson PTJ. Community ecology of invasions: direct and indirect effects of multiple invasive species on aquatic communities. Ecology. 2012;93(6):1254–61. PubMed PMID: WOS:000305296600002. 22834365 10.1890/11-1821.1

[2] Tennessen JB, Parks SE, Tennessen TP, Langkilde T. Raising a racket: invasive species compete acoustically with native treefrogs. Anim Behav. 2016;114:53–61. 10.1016/j.anbehav.2016.01.021 PubMed PMID: WOS:000373375100009.

[3] Vitousek PM. Biological invasions and ecosystem processes: towards an integration of population biology and ecosystem studies In: Samson FB, Knopf FL, editors. Ecosystem management: Selected readings. New York: Springer; 1990 p. 183–91.

[4] Ehrenfeld JG. A Different Perspective On Plant Invasions. Ecology. 2000;81(2):600–1. 10.1890/0012-9658(2000)081[0600:ADPOPI]2.0.CO;2

[5] Martinson HM, Fagan WF. Trophic disruption: a meta-analysis of how habitat fragmentation affects resource consumption in terrestrial arthropod systems. Ecol Lett. 2014;17(9):1178–89. 10.1111/ele.12305 PubMed PMID: WOS:000340406200014. 24866984

[6] Armstrong DP, Gorman N, Pike R, Kreigenhofer B, McArthur N, Govella S, et al Strategic rat control for restoring populations of native species in forest fragments. Conserv Biol. 2014;28(3):713–23. 10.1111/cobi.12256 PubMed PMID: WOS:000335809300016. 24617847

[7] Didham RK, Tylianakis JM, Gemmell NJ, Rand TA, Ewers RM. Interactive effects of habitat modification and species invasion on native species decline. Trends Ecol Evol. 2007;22(9):489–96. 10.1016/j.tree.2007.07.001 PubMed PMID: WOS:000249249100010. 17673330

[8] Didham RK, Tylianakis JM, Hutchison MA, Ewers RM, Gemmell NJ. Are invasive species the drivers of ecological change? Trends Ecol Evol. 2005;20(9):470–4. 10.1016/j.tree.2005.07.006 PubMed PMID: WOS:000232128300003. 16701420

[9] Denno R, Finke D, Langellotto G. Direct and indirect effects of vegetation structure and habitat complexity on predator–prey and predator–predator interactions In: Barbosa J, Castellanos I, editors. Ecology of Predator-Prey Interaction. New York: Oxford University Press; 2005 p. 211–39.

[10] Post DM, Pace ML, Hairston NG. Ecosystem size determines food-chain length in lakes. Nature. 2000;405(6790):1047–9. 10.1038/35016565 10890443

[11] Rayner MJ, Hauber ME, Imber MJ, Stamp RK, Clout MN. Spatial heterogeneity of mesopredator release within an oceanic island system. Proc Natl Acad Sci U S A. 2007;104(52):20862–5. 10.1073/pnas.0707414105 PubMed PMID: WOS:000252077400045. 18083843 PMC2409232

[12] Schmitz OJ. Predator diversity and trophic interactions. Ecology. 2007;88(10):2415–26. 10.1890/06-0937.1 PubMed PMID: WOS:000250714200001. 18027743

[13] Diehl S, Feissel M. Intraguild prey suffer from enrichment of their resources: A microcosm experiment with ciliates. Ecology. 2001;82(11):2977–83. 10.1890/0012-9658(2001)082[2977:ipsfeo]2.0.co;2 PubMed PMID: WOS:000172139500001.

[14] Holdaway R. Introduced predators and avifaunal extinction in New Zealand In: McPhee R, editor. Extinctions in Near Time: Causes, Contexts, and Consequences. Boston, MA: Springer; 1999 p. 189–238.

[15] Tylianakis JM, Didham RK, Bascompte J, Wardle DA. Global change and species interactions in terrestrial ecosystems. Ecol Lett. 2008;11(12):1351–63. 19062363 10.1111/j.1461-0248.2008.01250.x

[16] Klose K, Cooper SD. Contrasting effects of an invasive crayfish (*Procambarus clarkii*) on two temperate stream communities. Freshw Biol. 2012;57(3):526–40. 10.1111/j.1365-2427.2011.02721.x PubMed PMID: WOS:000299102000008.

[17] Polis GA, Sears ALW, Huxel GR, Strong DR, Maron J. When is a trophic cascade a trophic cascade? Trends Ecol Evol. 2000;15(11):473–5. 10.1016/s0169-5347(00)01971-6 PubMed PMID: WOS:000165122400025. 11050351

[18] Thompson J, Lugo AE, Thomlinson J. Land use history, hurricane disturbance, and the fate of introduced species in a subtropical wet forest in Puerto Rico. Plant Ecol. 2007;192(2):289–301. 10.1007/s11258-007-9318-5 PubMed PMID: WOS:000249270300013.

[19] Turner MG. Landscape ecology: What is the state of the science? Annu Rev Ecol Evol Syst. 2005;36(1):319–44. 10.1146/annurev.ecolsys.36.102003.152614 PubMed PMID: WOS:000234684900014.

[20] Vandermeer J. Oscillating populations and biodiversity maintenance. Bioscience. 2006;56(12):967–75. 10.1641/0006-3568(2006)56[967:opabm]2.0.co;2 PubMed PMID: WOS:000242795000006.

[21] Schmitz OJ, Hawlena D, Trussell GC. Predator control of ecosystem nutrient dynamics. Ecol Lett. 2010;13(10):1199–209. 10.1111/j.1461-0248.2010.01511.x 20602626

[22] Wardle DA, Bellingham PJ, Bonner KI, Mulder CPH. Indirect effects of invasive predators on litter decomposition and nutrient resorption on seabird-dominated islands. Ecology. 2009;90(2):452–64. 10.1890/08-0097.1 19323229

[23] Wilson EE, Wolkovich EM. Scavenging: how carnivores and carrion structure communities. Trends Ecol Evol. 2011;26(3):129–35. 10.1016/j.tree.2010.12.011 PubMed PMID: WOS:000288478100008. 21295371

[24] Strauss SY, Lau JA, Carroll SP. Evolutionary responses of natives to introduced species: what do introductions tell us about natural communities? Ecol Lett. 2006;9(3):354–71. 10.1111/j.1461-0248.2005.00874.x PubMed PMID: WOS:000235099600013. 16958902

[25] Losos JB, Schoener TW, Spiller DA. Predator-induced behaviour shifts and natural selection in field-experimental lizard populations. Nature. 2004;432(7016):505–8. 10.1038/nature03039 PubMed PMID: WOS:000225322100047. 15565155

[26] Stone PA, Snell HL, Snell HM. Behavioral diversity as biological diversity—introduced cats and lava lizard wariness. Conserv Biol. 1994;8(2):569–73. 10.1046/j.1523-1739.1994.08020569.x PubMed PMID: WOS:A1994NN16500031.

[27] Tolson PJ. Critical habitat, predator pressures, and the management of *Epicrates monensis* (Serpentes: Boidae) on the Puerto Rico bank: A multivariate analysis Flagstaff, AZ: USDA, 1988.

[28] O'Dowd DJ, Green PT, Lake PS. Invasional 'meltdown' on an oceanic island. Ecol Lett. 2003;6(9):812–7. PubMed PMID: ISI:000185095600004.

[29] Courchamp F, Chapuis JL, Pascal M. Mammal invaders on islands: impact, control and control impact. Biological Reviews. 2003;78(3):347–83. PubMed PMID: ISI:000185687200001. 14558589 10.1017/s1464793102006061

[30] Kier G, Kreft H, Lee TM, Jetz W, Ibisch PL, Nowicki C, et al A global assessment of endemism and species richness across island and mainland regions. Proc Natl Acad Sci U S A. 2009;106(23):9322–7. 10.1073/pnas.0810306106 19470638 PMC2685248

[31] Denslow JS. Weeds in paradise: thoughts on the invasibility of tropical islands. Ann Missouri Bot Gard. 2003;90(1):119–27.

[32] Freed LA, Cann RL. Negative Effects of an Introduced Bird Species on Growth and Survival in a Native Bird Community. Curr Biol. 2009;19(20):1736–40. 10.1016/j.cub.2009.08.044 PubMed PMID: WOS:000271498400030. 19765990

[33] Camp RJ, Pratt TK, Gorresen PM, Woodworth BL, Jeffrey JJ. Hawaiian forest bird trends: Using log-linear models to assess long-term trends is supported by model diagnostics and assumptions (reply to Freed and Cann 2013). Condor. 2014;116(1):97–101. 10.1650/condor-13-089.1 PubMed PMID: WOS:000335611300010.

[34] Wilson EE, Holway DA. Multiple mechanisms underlie displacement of solitary Hawaiian Hymenoptera by an invasive social wasp. Ecology. 2010;91(11):3294–302. 10.1890/09-1187.1 21141190

[35] Banko PC, Oboyski PT, Slotterback JW, Dougill SJ, Goltz DM, Johnson L, et al Availability of food resources, distribution of invasive species, and conservation of a Hawaiian bird along a gradient of elevation. Journal of Biogeography. 2002;29(5–6):789–808. PubMed PMID: ISI:000176652300019.

[36] Hanna C, Foote D, Kremen C. Competitive impacts of an invasive nectar thief on plant-pollinator mutualisms. Ecology. 2014;95(6):1622–32. PubMed PMID: WOS:000337218500020. 25039226 10.1890/13-1276.1

[37] Atkinson IAE. The spread of commensal species of *Rattus* to oceanic islands and their effects on island avifaunas. Conservation of Island Birds: Case Studies for the Management of Threatened Island Species 1985;International Council for Bird Preservation Technical Publication 3:35–81.

[38] Martin J-L, Thibault J-C, Bretagnolle V. Black rats, island characteristics, and colonial nesting birds in the Mediterranean: consequences of an ancient introduction. Conserv Biol. 2000;14(5):1452–66. 10.1046/j.1523-1739.2000.99190.x

[39] Drever MC, Harestad AS. Diets of Norway rats, *Rattus norvegicus*, on Langara Island, Queen Charlotte Islands, British Columbia: implications for conservation of breeding seabirds. Can Field Nat. 1998;112(4):676–83.

[40] Amarasekare P. Potential impact of mammalian nest predators on endemic forest birds of Western Mauna Kea, Hawaii. Conserv Biol. 1993;7(2):316–24. 10.1046/j.1523-1739.1993.07020316.x

[41] Amarasekare P. Ecology of introduced small mammals on Western Mauna Kea, Hawaii. J Mammal. 1994;75(1):24–38. 10.2307/1382233

[42] Levy S. Getting the drop on Hawaiian invasives. BioScience. 2003;53(8):694–9.

[43] Lindsey G, Hess S, Campbell E III, Sugihara RT. Small mammals as predators and competitors Conservation Biology of Hawaiian Forest Birds. New Haven, CT: Yale University Press; 2009 p. 274–92.

[44] Atkinson I. A reassessment of factors, particularly *Rattus rattus* L., that influenced the decline of endemic forest birds in the Hawaiian Islands. Pac Sci. 1977;31:109–33.

[45] Clark DA. Foraging behavior of a vertebrate omnivore (*Rattus rattus*): meal structure, sampling, and diet breadth. Ecology. 1982;63(3):763–72. 10.2307/1936797

[46] Cole FR, Loope LL, Medeiros AC, Howe CE, Anderson LJ. Food habits of introduced rodents in high-elevation shrubland of Haleakala National Park, Maui, Hawai'i. Pac Sci. 2000;54(4):313–29. doi: 10125/1659.

[47] Shiels AB, Pitt WC, Sugihara RT, Witmer GW. Biology and Impacts of Pacific Island Invasive Species. 11. *Rattus rattus*, the Black Rat (Rodentia: Muridae). Pac Sci. 2014;68(2):145–84. 10.2984/68.2.1 PubMed PMID: WOS:000336238400001.

[48] Sugihara RT. Abundance and diets of rats in two native Hawaiian forests. Pac Sci. 1997;51:189–98.

[49] Towns DR, Atkinson IAE, Daugherty CH. Have the harmful effects of introduced rats on islands been exaggerated? Biol Inv. 2006;8(4):863–91. 10.1007/s10530-005-0421-z PubMed PMID: WOS:000238531200027.

[50] VanderWerf EA, Smith DG. Effects of alien rodent control on demography of the O'ahu 'Elepaio, an endangered Hawaiian forest bird. Pac Conserv Biol. 2002;8(2):73–81. PubMed PMID: BCI:BCI200300069375.

[51] MacArthur RH. Population ecology of some warblers of northeastern coniferous forests. Ecology. 1958;39(4):599–619. 10.2307/1931600 PubMed PMID: WOS:A1958WR63100004.

[52] Schoener T. Resource partitioning In: Kikkawa D, editor. Community ecology: patterns and processes. London, UK: Blackwell Scientific; 1986 p. 91–126.

[53] Berryman AA, Hawkins BA. The refuge as an integrating concept in ecology and evolution. Oikos. 2006;115(1):192–6. PubMed PMID: WOS:000240998300020.

[54] Harrington LA, Harrington AL, Yamaguchi N, Thom MD, Ferreras P, Windham TR, et al The impact of native competitors on an alien invasive: temporal niche shifts to avoid interspecific aggression? Ecology. 2009;90(5):1207–16. 10.1890/08-0302.1 PubMed PMID: WOS:000265816200005. 19537542

[55] VanderWerf EA, Mosher SM, Burt M, Taylor P, Sailer D. Variable efficacy of rat control in conserving O‘ahu ‘Elepaio populations In: Veitch C, Clout M, Towns D, editors. Island invasives: eradication and management. Gland, Switzerland: International Union for the Conservation of Nature; 2011 p. 124–30.

[56] Kovach T. Determinants of avian density across a fragmented landscape Hilo, HI: University of Hawai'i at Hilo; 2012.

[57] Vaughn NR, Asner GP, Giardina CP. Centennial impacts of fragmentation on the canopy structure of tropical montane forest. Ecol Appl. 2014;24(7):1638–50. 10.1890/13-1568.1 29210228

[58] Flaspohler DJ, Giardina CP, Asner GP, Hart P, Price J, Lyons CK, et al Long-term effects of fragmentation and fragment properties on bird species richness in Hawaiian forests. Biol Cons. 2010;143(2):280–8. 10.1016/j.biocon.2009.10.009

[59] Vaughn NR, Asner GP, Giardina CP. Long-term fragmentation effects on the distribution and dynamics of canopy gaps in a tropical montane forest. Ecosphere. 2015;6(12):1–15. 10.1890/ES15-00235.1

[60] Vafidis JO, Vaughan IP, Jones TH, Facey RJ, Parry R, Thomas RJ. The effects of supplementary food on the breeding performance of Eurasian reed warblers *Acrocephalus scirpaceus*; Implications for climate change impacts. PLoS One. 2016;11(7). 10.1371/journal.pone.0159933 PubMed PMID: WOS:000381516100075. 27467171 PMC4965089

[61] Vafidis JO, Vaughan IP, Jones TH, Facey RJ, Parry R, Thomas RJ. Habitat use and body mass regulation among warblers in the Sahel region during the non-breeding season. PLoS One. 2014;9(11). 10.1371/journal.pone.0113665 PubMed PMID: WOS:000349145400076. 25426716 PMC4245214

[62] King RS, Wrubleski DA. Spatial and diel availability of flying insects as potential duckling food in prairie wetlands. Wetlands. 1998;18(1):100–14. PubMed PMID: WOS:000072925900012.

[63] Gruner DS. Attenuation of top-down and bottom-up forces in a complex terrestrial community. Ecology. 2004;85(11):3010–22. doi: doi:i0012-9658-085-11-3010. PubMed PMID: ISI:000225263500011.

[64] Canaday CL. Comparison of insect fauna captured in six different trap types in a Douglas-fir forest. The Canadian Entomologist. 1987;119(12):1101–8. 10.4039/Ent1191101-12

[65] Gruner DS. Regressions of length and width to predict arthropod biomass in the Hawaiian Islands University of Hawai'i Press; 2003.

[66] Banko P, Banko W. Evolution and ecology of food exploitation In: Pratt TK, Atkinson C, Banko P, Jacobi J, Woodworth B, editors. Conservation Biology of Hawaiian Forest Birds. New Haven: Yale University Press; 2009 p. 159–93.

[67] Baldwin P. Annual Cycle, Environment and Evolution in the Hawaiian Honeycreepers (Aves Depaniidae). U Calif Pub Zool 1953;52:285–398.

[68] Knowlton JL, Flaspohler DJ, Paxton EH, Fukami T, Giardina CP, Gruner DS, et al Movement behavior of native Hawaiian birds in a naturally fragmented landscape. J Avian Biol. 2017;48(7):921–31. 10.1111/jav.00924

[69] Latta SC, Wunderle JM. The composition and foraging ecology of mixed-species flocks in pine forests of Hispaniola. Condor. 1996;98(3):595–607. 10.2307/1369572 PubMed PMID: WOS:A1996VG52500014.

[70] Vaughn N, Asner G, Giardina C. Polar grid fraction as an estimator of forest canopy structure using airborne LiDAR. Remote Sensing Letters. 2013;34:7464–73. doi: citeulike-article-id:12239613.

[71] Barton K. MuMIn: Multi-Model Inference. R package version 1.40.4. R package version 1.40.4 ed: https://CRAN.R-project.org/package=MuMIn; 2018.

[72] R Core Team. R: A language and environment for statistical computing Vienna, Austria: R Foundation for Statistical Computing; 2018 p. http://www.R-project.org/.

[73] Kuznetsova A, Brockhoff PB, Christensen RHB. lmerTest: Tests for random and fixed effects for linear mixed effect models (lmer objects of lme4 package). R package version 1.2–1. ed: http://CRAN.R-project.org/package=lmerTest; 2013.

[74] Bates D, Maechler M, Bolker B. lme4: Linear mixed-effects models using S4 classes. R package version 0.999999–2 ed: http://CRAN.R-project.org/package=lme4; 2013.

[75] Franks A. mer-utils.R. 2014. p. https://github.com/aufrank/R-hacks/blob/master/mer-utils.R.

[76] Tremblay A, Ransijn J. LMERConvenienceFunctions: A suite of functions to back-fit fixed effects and forward-fit random effects, as well as other miscellaneous functions. 2015. p. http://CRAN.R-project.org/package=LMERConvenienceFunctions.

[77] De Rosario-Martinez H. phia: Post-Hoc Interaction Analysis. R package version 0.2–1. https://CRAN.R-project.org/package=phia. 2015.

[78] Burnham KP, Anderson DR. Model selection and multimodel inference: a practical information-theoretic approach 2nd ed New York, New York, USA: Springer-Verlag; 2002.

[79] Banko PC, Peck RW, Brinck KW, Leonard DL. Richness, diversity and similarity of arthropod prey consumed by a community of Hawaiian forest birds Hilo, HI: Hawai'i Cooperative Studies Unit, 2015 Contract No.: Technical Report HCSU-066.

[80] Manhaes MA, Dias MM, Lima ALC. Feeding resource partitioning between two understorey insectivorous birds in a fragment of Neotropical cloud forest. Braz J Biol. 2015;75(4):S176–S83. 10.1590/1519-6984.09114 PubMed PMID: WOS:000367098400021. 26602358

[81] Razeng E, Watson DM. What do declining woodland birds eat? A synthesis of dietary records. Emu. 2012;112(2):149–56. 10.1071/mu11099 PubMed PMID: WOS:000304356100009.

[82] Hagar JC, Dugger KM, Starkey EE. Arthropod prey of Wilson's warblers in the understory of douglas fir forests. Wilson J Ornithol. 2007;119(4):533–46. 10.1676/06-056.1 PubMed PMID: WOS:000252113600002.

[83] Crisol-Martinez E, Moreno-Moyano LT, Wormington KR, Brown PH, Stanley D. Using next-generation sequencing to contrast the diet and explore pest-reduction services of sympatric bird species in macadamia orchards in Australia. PLoS One. 2016;11(3). 10.1371/journal.pone.0150159 PubMed PMID: WOS:000371434500109. 26930484 PMC4773005

[84] Woodworth B, Pratt T. Life history and demography In: Pratt T, Atkinson CT, Banko P, Jacobi J, Woodworth B, editors. Conservation Biology of Hawaiian Forest Birds. New Haven: Yale University Press; 2009 p. 194–223.

[85] Pitt WC. Effects of multiple vertebrate predators on grasshopper habitat selection: trade-offs due to predation risk, foraging, and thermoregulation. Evol Ecol. 1999;13(5):499–515. 10.1023/a:1006792726166 PubMed PMID: WOS:000089346300005.

[86] VanderWerf EA. Rodent control decreases predation on artificial nests in O‘ahu ‘elepaio habitat. J Field Ornithol. 2001;72(3):448–57. 10.1648/0273-8570(2001)072[0448:rcdpoa]2.0.co;2

[87] Shiels A, Flores C, Khamsing A, Krushelnycky P, Mosher S, Drake D. Dietary niche differentiation among three species of invasive rodents (*Rattus rattus*, *R*. *exulans*, *Mus musculus*). Biol Inv. 2013;15(5):1037–48. 10.1007/s10530-012-0348-0

[88] Poulin B, Lefebvre G, McNeil R. Characteristics of feeding guilds and variation in diets of bird species of three adjacent tropical sites. Biotropica. 1994;26(2):187–97. 10.2307/2388808

[89] Sheppard SK, Harwood JD. Advances in molecular ecology: tracking trophic links through predator-prey food-webs. Funct Ecol. 2005;19(5):751–62. PubMed PMID: ISI:000232439600001.

[90] Symondson WOC. Molecular identification of prey in predator diets. Mol Ecol. 2002;11(4):627–41. PubMed PMID: ISI:000175250300001. 11972753 10.1046/j.1365-294x.2002.01471.x

[91] Massaro M, Starling-Windhof A, Briskie JV, Martin TE. Introduced mammalian predators induce behavioural changes in parental care in an endemic New Zealand bird. PLoS One. 2008;3(6):e2331 10.1371/journal.pone.0002331 18523640 PMC2396284

[92] Vanderwerf EA. Evolution of nesting height in an endangered Hawaiian forest bird in response to a non-native predator. Conserv Biol. 2012;26(5):905–11. 10.1111/j.1523-1739.2012.01877.x PubMed PMID: WOS:000308484500016. 22830652

[93] Burke DM, Nol E. Influence of food abundance, nest-site habitat, and forest fragmentation on breeding ovenbirds. Auk. 1998;115(1):96–104. PubMed PMID: WOS:000071487100011.

[94] Miller JR, Cale P. Behavioral mechanisms and habitat use by birds in a fragmented agricultural landscape. Ecol Appl. 2000;10(6):1732–48. 10.2307/2641235 PubMed PMID: WOS:000165680300013.

[95] Robinson SK. Another threat posed by forest fragmentation: Reduced food supply. Auk. 1998;115(1):1–3. PubMed PMID: WOS:000071487100001.

[96] Bregman TP, Sekercioglu CH, Tobias JA. Global patterns and predictors of bird species responses to forest fragmentation: Implications for ecosystem function and conservation. Biol Cons. 2014;169:372–83. 10.1016/j.biocon.2013.11.024 PubMed PMID: WOS:000333574400041.

[97] Gray MA, Baldauf SL, Mayhew PJ, Hill JK. The response of avian feeding guilds to tropical forest disturbance. Conserv Biol. 2007;21(1):133–41. 10.1111/j.1523-1739.2006.00557.x PubMed PMID: WOS:000244148800022. 17298519

[98] Ribon R, Simon JE, De Mattos GT. Bird extinctions in Atlantic forest fragments of the Vicosa region, southeastern Brazil. Conserv Biol. 2003;17(6):1827–39. 10.1111/j.1523-1739.2003.00377.x PubMed PMID: WOS:000186869700040.

[99] Ralph C, Fancy S. Demography and movements of 'Apapane and 'I'iwi in Hawaii. Condor. 1995:729–42.

[100] Lindsey G, Vanderwerf E, Baker H, Baker P. Hawai‘i 'Amakihi (*Hemignathus virens*) In: Poole A, editor. The birds of North America. New York: Cornell Lab of Ornithology; 1998.

[101] Maron JL, Estes JA, Croll DA, Danner EM, Elmendorf SC, Buckelew SL. An introduced predator alters Aleutian Island plant communities by thwarting nutrient subsidies. Ecological Monographs. 2006;76(1):3–24.

[102] Takimoto G, Spiller DA, Post DM. Ecosystem size, but not disturbance, determines food-chain length on islands of the Bahamas. Ecology. 2008;89(11):3001–7. 10.1890/07-1990.1 31766806

[103] Terborgh J, Lopez L, Nunez P, Rao M, Shahabuddin G, Orihuela G, et al Ecological meltdown in predator-free forest fragments. Science. 2001;294(5548):1923–6. 10.1126/science.1064397 PubMed PMID: WOS:000172465000058. 11729317

[104] Tingley R, Vallinoto M, Sequeira F, Kearney MR. Realized niche shift during a global biological invasion. Proc Natl Acad Sci U S A. 2014;111(28):10233–8. 10.1073/pnas.1405766111 PubMed PMID: WOS:000338985700052. 24982155 PMC4104887

[105] Guisan A, Petitpierre B, Broennimann O, Daehler C, Kueffer C. Unifying niche shift studies: insights from biological invasions. Trends Ecol Evol. 2014;29(5):260–9. 10.1016/j.tree.2014.02.009 PubMed PMID: WOS:000336113600005. 24656621

