# Supplementary material for: Vertical foraging shifts in Hawaiian forest birds in response to invasive rat removal
Source: PLoS One. 2018 Sep 24;13(9):e0202869. doi: 10.1371/journal.pone.0202869 (PMC6152863; doi:10.1371/journal.pone.0202869)

Supplemental Materials:

Vertical foraging shifts in Hawaiian forest birds in response to invasive rat removal

Erin E. Wilson Rankin[1,#a*¶], Jessie L. Knowlton[2,#b¶], Daniel S. Gruner[1], David J. Flaspohler[2], Christian P. Giardina[3], Devin R. Leopold[4], Anna Buckardt[2], William C. Pitt[5], Tadashi Fukami[4]

[1] Department of Entomology, University of Maryland, College Park, Maryland, United States of America

[2] School of Forest Resources and Environmental Science, Michigan Technological University, Houghton, Michigan, United States of America

[3] Institute of Pacific Islands Forestry, United States Department of Agriculture, United States Forest Service, Hilo, Hawaiʻi, United States of America

[4] Department of Biology, Stanford University, Stanford, California, United States of America

[5] Smithsonian Conservation Biology Institute, Smithsonian Institution, Front Royal, Virginia, United States of America

[#a] Current address: Department of Entomology, University of California Riverside, Riverside, California, United States of America

[#b] Current address: Department of Biology, Wheaton College, Boston, Massachusetts, United States of America

S1 Figure. Height of canopy increases with kipuka size. Canopy heights were averaged over all foraging observations by kipuka. Data are shown ± SE. Solid black line indicates line of best fit for treated (rat-removed) kipuka and dotted gray for untreated kipuka. Canopy height increased with kipuka size and did not differ between treatments (Table S2).

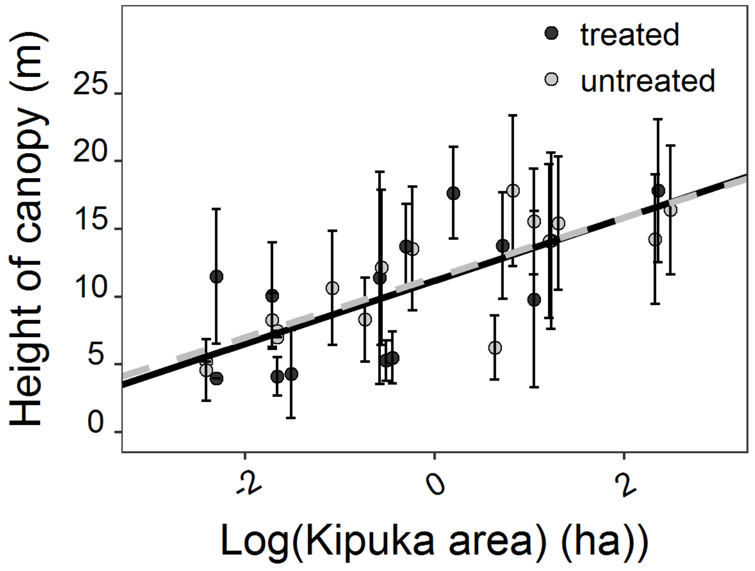

Supplement: S1 Fig — Canopy heights were averaged over all foraging observations by kīpuka. Data are shown ± SE. Solid black line indicates line of best fit for treated kīpuka and dotted gray for untreated kīpuka. Canopy height increased with kīpuka size and did not differ between treatments. (PDF) [file pone.0202869.s001.pdf]
