# Supplementary material for: Vertical foraging shifts in Hawaiian forest birds in response to invasive rat removal
Source: PLoS One. 2018 Sep 24;13(9):e0202869. doi: 10.1371/journal.pone.0202869 (PMC6152863; doi:10.1371/journal.pone.0202869)

Supplemental Materials:

Vertical foraging shifts in Hawaiian forest birds in response to invasive rat removal

Erin E. Wilson Rankin[1,#a*¶], Jessie L. Knowlton[2,#b¶], Daniel S. Gruner[1], David J. Flaspohler[2], Christian P. Giardina[3], Devin R. Leopold[4], Anna Buckardt[2], William C. Pitt[5], Tadashi Fukami[4]

[1] Department of Entomology, University of Maryland, College Park, Maryland, United States of America

[2] School of Forest Resources and Environmental Science, Michigan Technological University, Houghton, Michigan, United States of America

[3] Institute of Pacific Islands Forestry, United States Department of Agriculture, United States Forest Service, Hilo, Hawaiʻi, United States of America

[4] Department of Biology, Stanford University, Stanford, California, United States of America

[5] Smithsonian Conservation Biology Institute, Smithsonian Institution, Front Royal, Virginia, United States of America

[#a] Current address: Department of Entomology, University of California Riverside, Riverside, California, United States of America

[#b] Current address: Department of Biology, Wheaton College, Boston, Massachusetts, United States of America

S2 Figure. Mean foraging height of birds by rat treatment and horizontal foraging position Foraging height was unaffected by horizontal foraging position of the bird within the canopy. Different letters indicate significance at $p < 0.01$ in post-hoc tests.

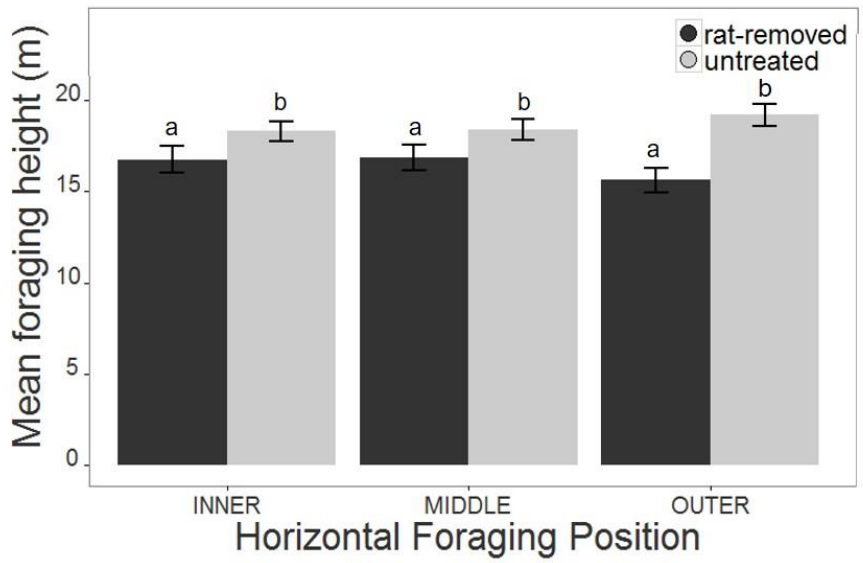

Supplement: S2 Fig — Foraging height was unaffected by horizontal foraging position of the bird within the canopy. Different letters indicate significance at p < 0.01. (PDF) [file pone.0202869.s002.pdf]
