# Supplementary material for: Vertical foraging shifts in Hawaiian forest birds in response to invasive rat removal
Source: PLoS One. 2018 Sep 24;13(9):e0202869. doi: 10.1371/journal.pone.0202869 (PMC6152863; doi:10.1371/journal.pone.0202869)
Supplement: S1 Table — (PDF) [file pone.0202869.s003.pdf]

Supplemental Materials:

Vertical foraging shifts in Hawaiian forest birds in response to invasive rat removal

Erin E. Wilson Rankin[1,#a*¶], Jessie L. Knowlton[2,#b¶], Daniel S. Gruner[1], David J. Flaspohler[2], Christian P. Giardina[3], Devin R. Leopold[4], Anna Buckardt[2], William C. Pitt[5], Tadashi Fukami[4]

[1] Department of Entomology, University of Maryland, College Park, Maryland, United States of America

[2] School of Forest Resources and Environmental Science, Michigan Technological University, Houghton, Michigan, United States of America

[3] Institute of Pacific Islands Forestry, United States Department of Agriculture, United States Forest Service, Hilo, Hawaiʻi, United States of America

[4] Department of Biology, Stanford University, Stanford, California, United States of America

[5] Smithsonian Conservation Biology Institute, Smithsonian Institution, Front Royal, Virginia, United States of America

[#a] Current address: Department of Entomology, University of California Riverside, Riverside, California, United States of America

[#b] Current address: Department of Biology, Wheaton College, Boston, Massachusetts, United States of America

S1 Table: GLMM Model Selection

The following are the model selection results for all statistical models. We reported the best model in S2-S7 Tables and in the text. Random effects are indicated by (1|random effect) notation.

Model	K	logLik	AICc	∆AICc	Weight
Kipuka characteristics and tree height					
log(Area_ha) + Rat_Removal + (1|Year) + (1|Kipuka) + (1|OBSERVER)	7	-1510.39	3034.99	0.000	0.6895
log(Area_ha) + (1|Year) + (1|Kipuka) + (1|OBSERVER)	6	-1512.21	3036.59	1.598	0.3102
Rat_Removal + (1|Year) + (1|Kipuka) + (1|OBSERVER)	6	-1519.97	3052.11	17.116	0.001
(1|Year) + (1|Kipuka) + (1|OBSERVER)	5	-1522.33	3054.78	19.788	0.000
Proportion of arthropod biomass					
Rat_Removal * Trap_height + (1|Kipuka)	7	-859.26	1733.21	0.000	0.271
Rat_Removal :Trap_height + Trap_height + (1|Kipuka)	7	-859.26	1733.21	0.000	0.271
Rat_Removal + Rat_Removal:Trap_height + (1|Kipuka)	7	-859.26	1733.21	0.000	0.271
Rat_Removal * Trap_height + log(Area_ha) + (1|Kipuka)	8	-859.22	1735.35	2.134	0.093
Rat_Removal + Rat_Removal:Trap_height + log(Area_ha) + (1|Kipuka)	8	-859.22	1735.35	2.134	0.093
Rat_Removal + Trap_height + log(Area_ha) + (1|Kipuka)	6	-870.17	1752.85	19.636	0.000
(1|Kipuka)	2	-889.47	1783.01	49.8	0
Proportion of vertical foraging space occupied (canopy utilization by birds)					
Diet * Rat_Removal + (1 | Year) + (1 | Kipuka)	8	-299.400	616.1093	0.000	0.352
Diet:Rat_Removal + Diet + log(Area_ha) + (1 | Year) + (1 | Kipuka)	9	-298.996	617.6438	1.534	0.163
Diet * Rat_Removal + log(Area_ha) + (1 | Year) + (1 | Kipuka)	9	-298.996	617.6438	1.534	0.163
Diet:Rat_Removal + Rat_Removal + log(Area_ha) + (1 | Year) + (1 | Kipuka)	9	-298.996	617.6438	1.534	0.163
Diet + Rat_Removal + log(Area_ha) + (1 | Year) + (1 | Kipuka)	7	-301.458	617.9258	1.816	0.142
Diet + log(Area_ha) + (1 | Year) + (1 | Kipuka)	6	-304.780	622.31	6.201	0.016
Rat_Removal + log(Area_ha) + (1 | Year) + (1 | Kipuka)	5	-316.993	644.5162	28.407	0.000
(1 | Year) + (1 | Kipuka)	3	-319.83	645.88	29.77	0.000
Foraging heights of Hawaiian forest birds by bird species					
Total arth biomass + Total arth biomass:Rat_Removal + log(Area_ha) + SPECIES + (1|Year) + (1|Kipuka) + (1|OBSERVER)	13	161.41	-296.12	0.000	0.731
Total arth biomass * Rat_Removal + log(Area_ha) + SPECIES + (1|Year) + (1|Kipuka) + (1|OBSERVER)	14	161.47	-294.17	2.003	0.269
Total arth biomass * Rat_Removal + log(Area_ha) + (1|Year) + (1|Kipuka) + (1|OBSERVER)	9	144.64	-270.94	25.182	0.000
Total arth biomass * Rat_Removal + SPECIES + (1|Year) + (1|Kipuka) + (1|OBSERVER)	13	144.86	-263.01	33.111	0.000
Total arth biomass + log(Area_ha) + SPECIES + (1|Year) + (1|Kipuka) + (1|OBSERVER)	12	114.51	-204.41	91.714	0.000
Total arth biomass + Rat_Removal + log(Area_ha) + SPECIES + (1|Year) + (1|Kipuka) + (1|OBSERVER)	13	114.68	-202.65	93.471	0.000
Rat_Removal + log(Area_ha) + SPECIES + (1|Year) + (1|Kipuka) + (1|OBSERVER)	12	81.62	-138.62	157.494	0.000
(1|Year) + (1|Kipuka) + (1|OBSERVER)					
Behavior impacts – data limited to 2012 when had behavior observations data					
Total arth biomass * Rat_Removal + log(Area_ha) + SPECIES + (1|Kipuka) + (1|OBSERVER)	14	-68.05	165.49	0.000	0.83
Total arth biomass * Rat_Removal + log(Area_ha) + SPECIES + Foraging.behavior + (1|Kipuka) + (1|OBSERVER)	20	-63.11	169.03	3.54	0.14
Total arth biomass * Rat_Removal + log(Area_ha) + SPECIES + Substrate.type + (1|Kipuka) + (1|OBSERVER)	19	-66.46	173.46	7.97	0.02

Total arth biomass * Rat_Removal + log(Area_ha) + SPECIES + Foraging.behavior + Horizontal.position + Foliage.density + (1|Kipuka) + (1|OBSERVER)	23	-62.79	175.31	9.83	0.01
Total arth biomass * Rat_Removal + log(Area_ha) + SPECIES + Foraging.behavior + Substrate.type + (1|Kipuka) + (1|OBSERVER)	25	-61.41	177.26	11.77	0.000
Total arth biomass * Rat_Removal + log(Area_ha) + SPECIES + Foraging.behavior + Foliage.density + Substrate.type + (1|Kipuka) + (1|OBSERVER)	26	-61.11	179.01	13.53	0.000
Total arth biomass * Rat_Removal + log(Area_ha) + SPECIES + Horizontal.position + Foliage.density + Substrate.type + (1|Kipuka) + (1|OBSERVER)	22	-66.12	179.65	14.17	0.000
Total arth biomass * Rat_Removal + log(Area_ha) + Foraging.behavior + Horizontal.position + Foliage.density + Substrate.type + (1|Kipuka) + (1|OBSERVER)	22	-66.70	180.82	15.33	0.000
Total arth biomass:Rat_Removal + Total arth biomass + log(Area_ha) + SPECIES + Foraging.behavior + Horizontal.position + Foliage.density + Substrate.type + (1|Kipuka) + (1|OBSERVER)	27	-61.02	181.22	15.73	0.000
Total arth biomass * Rat_Removal + log(Area_ha) + SPECIES + Foraging.behavior + Horizontal.position + Substrate.type + (1|Kipuka) + (1|OBSERVER)	27	-61.25	181.68	16.20	0.000
Total arth biomass:Rat_Removal + Rat_Removal + log(Area_ha) + SPECIES + Foraging.behavior + Horizontal.position + Foliage.density + Substrate.type + (1|Kipuka) + (1|OBSERVER)	28	-60.94	183.46	17.97	0.000
Total arth biomass * Rat_Removal + log(Area_ha) + SPECIES + Foraging.behavior + Horizontal.position + Foliage.density + Substrate.type + (1|Kipuka) + (1|OBSERVER)	28	-60.94	183.46	17.97	0.000
Total arth biomass + Rat_Removal + log(Area_ha) + SPECIES + Foraging.behavior + Horizontal.position + Foliage.density + Substrate.type + (1|Kipuka) + (1|OBSERVER)	27	-65.26	189.69	24.21	0.000
Total arth biomass * Rat_Removal + SPECIES + Foraging.behavior + Horizontal.position + Foliage.density + Substrate.type + (1|Kipuka) + (1|OBSERVER)	27	-73.54	206.26	40.78	0.000
(1|Kipuka) + (1|OBSERVER)	4	-109.75	227.62	62.13	0.000
