# Supplementary material for: Vertical foraging shifts in Hawaiian forest birds in response to invasive rat removal
Source: PLoS One. 2018 Sep 24;13(9):e0202869. doi: 10.1371/journal.pone.0202869 (PMC6152863; doi:10.1371/journal.pone.0202869)
Supplement: S2 Table — (PDF) [file pone.0202869.s004.pdf]

Appendix: GLMM Model Results

The following are the model average outputs from model.avg call of the MUMIn package in R software, as described in the text. For each averaged model, we report the parameter estimates, their standard errors, Z values and corresponding p-values. Bolded entries in the tables were reported in the text. Asterisks indicate level of significance: * p < 0.05, ** p < 0.01, *** p < 0.001.

S2 Table. Kipuka characteristics and tree height.

	**Estimate**	**Std. Error**	**Adjusted SE**	**z value**	**Pr(>|z|)**	
(Intercept)	0	0	0	NA	NA	
log(Area_ha)	0.41897	0.0786	0.07879	5.318	1.10E-07	***
Rat_Removaluntreated	0.11706	0.09047	0.09068	1.291	0.197	

* Rat_Removal: categorical variable with 2 levels ("untreated" used as reference level).

**Relative variable importance:**	**Rat_Removal**	**log(Area_ha)**
Importance:	1.0	0.69
N containing models:	**2**	**2**
