# Supplementary material for: Vertical foraging shifts in Hawaiian forest birds in response to invasive rat removal
Source: PLoS One. 2018 Sep 24;13(9):e0202869. doi: 10.1371/journal.pone.0202869 (PMC6152863; doi:10.1371/journal.pone.0202869)
Supplement: S3 Table — (PDF) [file pone.0202869.s005.pdf]

Appendix: GLMM Model Results

The following are the model average outputs from model.avg call of the MUMIn package in R software, as described in the text. For each averaged model, we report the parameter estimates, their standard errors, Z values and corresponding p-values. Bolded entries in the tables were reported in the text. Asterisks indicate level of significance: * p < 0.05, ** p < 0.01, *** p < 0.001.

S3 Table. Proportion of arthropod biomass.

	**Estimate**	**Std. Error**	**Adjusted SE**	**z value**	**Pr(>|z|)**	
(Intercept)	0	0	0	NA	NA	
Trap_heightmid	-0.284	0.4206	0.4238	0.67	0.50279	
Trap_heighthigh	-1.1949	0.4438	0.4471	2.672	0.00753	**
Rat_Removaluntreated:Trap_heightlow	-0.3952	0.2988	0.3011	1.312	0.18935	
Rat_Removaluntreated:Trap_heightmid	-0.6393	0.3609	0.3631	1.761	0.07825	.
Rat_Removaluntreated:Trap_heighthigh	1.035	0.5908	0.5922	1.748	0.08052	.
Rat_Removaluntreated	-0.332	0.2515	0.2534	1.31	0.19016	
Rat_Removaltreated:Trap_heightmid	-0.1354	0.2007	0.2022	0.67	0.50299	
Rat_Removaltreated:Trap_heighthigh	-0.599	0.2224	0.2241	2.673	0.00752	**
log(Area_ha)	-0.0388	0.1457	0.1468	0.264	0.79157	

**Relative variable importance:**	**Rat_Removal:Trap_height**	**Rat_Removal**	**Trap_height**	**log(Area_ha)**
Importance:	1	0.73	0.64	0.19
N containing models:	5	5	4	3

*Trap height: categorical variable with 3 levels ("low" used as reference level). Rat_Removal: categorical variable with 2 levels ("untreated" used as reference level).
