# Supplementary material for: Vertical foraging shifts in Hawaiian forest birds in response to invasive rat removal
Source: PLoS One. 2018 Sep 24;13(9):e0202869. doi: 10.1371/journal.pone.0202869 (PMC6152863; doi:10.1371/journal.pone.0202869)
Supplement: S4 Table — (PDF) [file pone.0202869.s006.pdf]

Appendix: GLMM Model Results

The following are the model average outputs from model.avg call of the MUMIn package in R software, as described in the text. For each averaged model, we report the parameter estimates, their standard errors, Z values and corresponding p-values. Bolded entries in the tables were reported in the text. Asterisks indicate level of significance: * $p < 0.05$, ** $p < 0.01$, *** $p < 0.001$.

S4 Table. Proportion of vertical foraging space occupied (canopy utilization by birds).

	**Estimate**	**Std. Error**	**Adjusted SE**	**z value**	**Pr(>|z|)**	
(Intercept)	0	0	0	NA	NA	
DietInsects	0.16342	0.06085	0.06149	2.658	0.00786	**
DietNectar	0.31056	0.05869	0.05934	5.234	2.00E-07	***
Rat_Removaluntreated	-0.06219	0.14665	0.14748	0.422	0.67323	
DietInsects:Rat_Removaluntreated	-0.09785	0.08126	0.08179	1.196	0.23154	
DietNectar:Rat_Removaluntreated	-0.10173	0.1301	0.13047	0.78	0.43555	
log(Area_ha)	0.08923	0.10045	0.10156	0.879	0.3796	
DietFruit:Rat_Removaluntreated	-0.01153	0.06078	0.06145	0.188	0.85114	
DietInsects:Rat_Removaltreated	0.18433	0.06424	0.06495	2.838	0.00454	**
DietNectar:Rat_Removaltreated	0.35432	0.06704	0.06778	5.227	2.00E-07	***

* Rat_Removal: categorical variable with 2 levels ("untreated" used as reference level). Diet: categorical variable of 3 levels ("fruit eating" used as reference level).

**Relative variable importance:**	**Diet:Rat_Removal**	**Diet**	**Rat_Removal**	**log(Area_ha)**
Importance:	0.84	0.84	0.82	0.65
N containing models:	4	5	5	6
