# Supplementary material for: Vertical foraging shifts in Hawaiian forest birds in response to invasive rat removal
Source: PLoS One. 2018 Sep 24;13(9):e0202869. doi: 10.1371/journal.pone.0202869 (PMC6152863; doi:10.1371/journal.pone.0202869)
Supplement: S5 Table — (PDF) [file pone.0202869.s007.pdf]

Appendix: GLMM Model Results

The following are the model average outputs from model.avg call of the MUMIn package in R software, as described in the text. For each averaged model, we report the parameter estimates, their standard errors, Z values and corresponding p-values. Bolded entries in the tables were reported in the text. Asterisks indicate level of significance: * $p < 0.05$, ** $p < 0.01$, *** $p < 0.001$.

S5 Table. Foraging heights of Hawaiian forest birds by bird species.

	Estimate	Std. Error	Adjusted SE	z value	Pr(>|z|)	
(Intercept)	0	0	0	NA	NA	
Total arth biomass	-0.35296	0.02757	0.02764	12.77	< 2e-16	***
log(Area_ha)	0.95802	0.12376	0.12405	7.723	< 2e-16	***
SPECIESHAAM	-0.07876	0.02114	0.02119	3.718	0.000201	***
SPECIESHAEL	-0.07386	0.02021	0.02026	3.646	0.000266	***
SPECIESIIWI	-0.03093	0.02184	0.0219	1.413	0.157746	
SPECIESJAWE	0.05036	0.02147	0.02152	2.34	0.019267	*
SPECIESOMAO	-0.01965	0.02061	0.02066	0.951	0.341588	
Total arth biomass:Rat_Removaluntreated	0.30516	0.08103	0.08106	3.765	0.000167	***
Rat_Removaluntreated	-0.04915	0.14536	0.14571	0.337	0.735879	
Total arth biomass:Rat_Removaltreated	-0.33155	0.0259	0.02596	12.772	< 2e-16	***
Total arth biomass:Rat_Removaluntreated	0.30516	0.08103	0.08106	3.765	0.000167	***

*Rat_Removal: categorical variable with 2 levels ("untreated" used as reference level). SPECIES: categorical variable with 6 levels (Apapane="APAP" used as reference level).

Relative variable importance:	Total arth biomass:Rat_Removal	log(Area_ha)	SPECIES	Total arth biomass	Rat_Removal
Importance:	1	1	1	0.79	0.42
N containing models:	5	7	7	6	6
