# Supplementary material for: Vertical foraging shifts in Hawaiian forest birds in response to invasive rat removal
Source: PLoS One. 2018 Sep 24;13(9):e0202869. doi: 10.1371/journal.pone.0202869 (PMC6152863; doi:10.1371/journal.pone.0202869)
Supplement: S6 Table — (PDF) [file pone.0202869.s008.pdf]

Appendix: GLMM Model Results

The following are the model average outputs from model.avg call of the MUMIn package in R software, as described in the text. For each averaged model, we report the parameter estimates, their standard errors, Z values and corresponding p-values. Bolded entries in the tables were reported in the text. Asterisks indicate level of significance: * p < 0.05, ** p < 0.01, *** p < 0.001.

S6 Table. Post-hoc testing for Species (p-value adjustment method: fdr) of best model described in above Table S5. Red-billed Leiothrix (RBLE) were only observed in treated kipuka and thus were excluded from subsequent post-hoc testing. APAP: ʻApapane; HAAM: Hawaiʻi ʻAmakihi; HAEL: Hawai'I ʻelepaio; IIWI: ʻI'ʼiwi; JAWE: Japanese White-eye; OMAO: Omaʻo.

Comparison	Value	Chisq	Pr(>Chisq)
APAP-HAAM	0.06801	10.9046	0.0095926 **
APAP-HAEL	0.33529	18.7717	0.0002063 ***
APAP-IIWI	0.03842	3.4046	0.2656942
APAP-JAWE	-0.06527	4.0726	0.2614974
APAP-OMAO	0.08633	3.7395	0.2656942
HAAM-HAEL	0.26728	11.448	0.0078726 **
HAAM-IIWI	-0.02959	1.341	0.7405758
HAAM-JAWE	-0.13328	13.5705	0.0027572 **
HAAM-OMAO	0.01833	0.1501	0.7405758
HAEL-IIWI	-0.29687	14.0441	0.0023214 **
HAEL-JAWE	-0.40056	23.4447	1.928e-05 ***
HAEL-OMAO	-0.24895	7.8725	0.0351352 *
IIWI-JAWE	-0.10369	8.3706	0.0343198 *
IIWI-OMAO	0.04791	1.0394	0.7405758
JAWE-OMAO	0.15161	8.2459	0.0343198 *
