# Supplementary material for: Vertical foraging shifts in Hawaiian forest birds in response to invasive rat removal
Source: PLoS One. 2018 Sep 24;13(9):e0202869. doi: 10.1371/journal.pone.0202869 (PMC6152863; doi:10.1371/journal.pone.0202869)
Supplement: S7 Table — (PDF) [file pone.0202869.s009.pdf]

Appendix: GLMM Model Results

The following are the model average outputs from model.avg call of the MUMIn package in R software, as described in the text. For each averaged model, we report the parameter estimates, their standard errors, Z values and corresponding p-values. Bolded entries in the tables were reported in the text. Asterisks indicate level of significance: * p < 0.05, ** p < 0.01, *** p < 0.001.

S7 Table. Behavior impacts – data limited to 2012 when had behavior observations data.

	**Estimate**	**Std. Error**	**Adjusted SE**	**z value**	**Pr(>|z|)**	
(Intercept)	0	0	0	NA	NA	
Total arth biomass	-0.31947	0.04498	0.04516	7.074	< 2e-16	***
Rat_Removaluntreated	-0.05578	0.14363	0.14421	0.387	0.69893	
log(Area_ha)	0.84836	0.1295	0.13003	6.524	< 2e-16	***
SPECIESHAAM	-0.1177	0.03851	0.03867	3.044	0.00233	**
SPECIESHAEL	-0.01447	0.036	0.03614	0.4	0.68881	
SPECIESIIWI	-0.08922	0.03925	0.03941	2.264	0.02359	*
SPECIESJAWE	-0.02357	0.03914	0.0393	0.6	5.49E-01	
SPECIESOMAO	-0.01322	0.04243	0.0426	0.31	0.75632	
SPECIESRBLE	-0.01421	0.03792	0.03808	0.373	0.70897	
FORAG.METHODGLEAN/PROBE	0.03236	0.03751	0.03766	0.859	0.39023	
FORAG.METHODHANG	-0.0186	0.03757	0.03772	0.493	0.62191	
FORAG.METHODLEAP	-0.06241	0.04269	0.04287	1.456	0.14539	
FORAG.METHODPROBE	-0.08749	0.0382	0.03836	2.281	0.02255	*
FORAG.METHODREACH	-0.06607	0.03819	0.03835	1.723	0.08491	.
FORAG.METHODSALLY	-0.01763	0.03763	0.03778	0.467	0.64077	
HORIZ.POSITMIDDLE	-0.02599	0.04654	0.04673	0.556	0.57809	
HORIZ.POSITOUTER	-0.01931	0.04866	0.04886	0.395	0.69261	
FOL.DENS	-0.02402	0.04058	0.04074	0.59	0.55543	
match:Rat_Removaluntreated	0.19449	0.06982	0.07009	2.775	0.00552	**
SUBSTRATE.TYPEBRANCH	-0.05078	0.04066	0.04083	1.244	0.21352	
SUBSTRATE.TYPEFRUIT	0.01917	0.03827	0.03842	0.499	0.61782	
SUBSTRATE.TYPETRUNK	0.03475	0.03751	0.03767	0.923	0.3562	
SUBSTRATE.TYPETWIG	0.04453	0.04927	0.04947	0.9	0.36807	
SUBSTRATE.TYPEUnknown	-0.01	0.04591	0.0461	0.217	0.8282	
Total arth biomass:Rat_Removaltreated	-0.3083	0.04276	0.04294	7.179	< 2e-16	***

*Rat_Removal: categorical variable with 2 levels ("untreated" used as reference level). SPECIES: categorical variable with 7 levels (Apapane="APAP" used as reference level). FORAG.METHOD: categorical variable with 7 levels ("glean" used as reference variable). HORIZ.POSIT: categorical variable with 3 levels ("inner" used as reference level). SUBSTRATE.TYPE: categorical variable with 6 levels ("leaf" used as reference variable).

	log (Area ha)	Total arth biomass: Rat_Removal	Total arth biomass	Rat Removal	SPECIES	FORAG. METHOD	SUBSTRATE TYPE	FOL DENS	HORIZ POSIT
Importance:	1	1	1	1	1	0.15	0.02	0.01	0.01
N containing models:	13	13	13	13	13	11	11	9	9
